# Tempest in a teacup: An analysis of *p*-Hacking in organizational research

**DOI:** 10.1371/journal.pone.0281938

**Published:** 2023-02-24

**Authors:** Alisha Gupta, Frank Bosco

**Affiliations:** Department of Management and Entrepreneurship, School of Business, Virginia Commonwealth University, Richmond, VA, United States of America; University of Valencia: Universitat de Valencia, SPAIN

## Abstract

We extend questionable research practices (QRPs) research by conducting a robust, large-scale analysis of *p*-hacking in organizational research. We leverage a manually curated database of more than 1,000,000 correlation coefficients and sample sizes, with which we calculate exact *p*-values. We test for the prevalence and magnitude of *p-*hacking across the complete database as well as various subsets of the database according to common bivariate relation types in the organizational literature (e.g., attitudes-behaviors). Results from two analytical approaches (i.e., z-curve, critical bin comparisons) were consistent in both direction and significance in nine of 18 datasets. Critical bin comparisons indicated *p*-hacking in 12 of 18 subsets, three of which reached statistical significance. Z-curve analyses indicated *p*-hacking in 11 of 18 subsets, two of which reached statistical significance. Generally, results indicated that *p-*hacking is detectable but small in magnitude. We also tested for three predictors of *p-*hacking: Publication year, journal prestige, and authorship team size. Across two analytic approaches, we observed a relatively consistent positive relation between *p*-hacking and journal prestige, and no relationship between *p-*hacking and authorship team size. Results were mixed regarding the temporal trends (i.e., evidence for *p*-hacking over time). In sum, the present study of *p-*hacking in organizational research indicates that the prevalence of *p-*hacking is smaller and less concerning than earlier research has suggested.

## Introduction

Questionable research practices (QRPs; misrepresentation, inaccuracy, or bias at any part of the research process) are reportedly on the rise [1,2]. Commonly studied QRPs include hypothesizing after results are known (HARKing; [3,4]), contingent inclusion or exclusion of influential observations, and *p*-hacking [5]. Such so-called questionable research practices fall between ideal and worst behaviors [2] and appear to occur with higher frequency than more serious practices like fabrication, falsification, and plagiarism. Indeed, Xie et al. [6] report that 39.70% of researchers reported awareness of others who had used at least one QRP, a 39.15% increase from Fanelli’s [7] earlier estimate of 28.53%. Moreover, among 20 disciplines, economics and business presented with the highest rate of reporting a positive result, possibly due to QRP engagement [8]. Although regarded as potentially less detrimental, QRPs can nonetheless distort cumulative evidence (e.g., upwardly or downwardly biased meta-analytic summaries) [9]. Furthermore, QRPs can shape future research with respect to “the development of theory, evidence-based practice, and perceptions of the rigor and relevance of science” [10, p. 323]. Although in some fields the negative effects of QRPs on replicability is questioned [11], QRPs in organizational research are considered to be a threat [6].

In this study, we focus on a QRP called *p*-hacking, the manipulation of data or analyses to reach conventional thresholds for statistical significance (e.g., *p* < .05; [5]). We focus on *p*-hacking because of its harmful implications for future research. First, *p*-hacking can yield biased or misreported effect sizes. As a result, meta-analyses would not reflect the true distribution of effect sizes and would transmit that bias [12,13]. Relatedly, *p*-hacking results in a disproportionate amount of statistically significant studies published in the literature on any given relationship, which can yield incorrect inferences about that relationship for other purposes, such as hypothesis development, narrative reviews, or inductive inference [13,14]. Thus, considering the effects of *p-*hacking on our cumulative evidence, concern regarding their prevalence is warranted.

Given the problematic consequences of *p*-hacking, various approaches to detect and quantify *p*-hacking have been developed including case studies [15], z- and *p*-curve analyses [16–18], test-statistic distribution analysis [19], or critical bin comparison analyses [13]. Indeed, applications of these approaches have appeared in areas such as public administration [20], accounting [21,22], psychophysiology [23–25], medicine [26,27], behavioral ecology and evolution [28,29], and experimental psychology [30–33]. In all of these cases, evidence of *p*-hacking has been observed. Baum and Bromiley [34] studied *p-*hacking in management research, though their limited sampling frame (i.e., two journals over 16 years, excluding articles authored by students or three or more authors) places limits on its precision and generalizability. Additionally, this study likely suffered from lack of statistical power, perhaps leading the authors to use unconventionally large *p*-value bin widths. Thus, to our knowledge, there exists no large-scale, comprehensive investigation of *p*-hacking in organizational research. Such a study is warranted because *p*-hacking can have potentially adverse effects on the trustworthiness of our cumulative scientific knowledge base and the science-practice gap (e.g., [26]).

It is worth noting up-front that there exist a variety of statistics to which *p*-values are attached. Some are explicitly hypothesized; in organizational research, these statistics tend to represent higher-order effects (e.g., tests for mediation, moderation, model comparisons). Others are much simpler, such as zero-order correlations reported in tables. (The term zero-order correlation is common to organizational research and refers to a correlation coefficient to which no statistical control is applied). Hypothesized higher-order relations are indeed important findings, but organizational science meta-analysts rarely accumulate them [35]. This is because relatively few primary studies propose and test the same complex hypotheses under the same constraints (e.g., using the same control variables). So, which is the appropriate sampling frame for a large-scale test of *p*-hacking in the cumulative knowledge of organizational research? In the present study, as in earlier studies of *p*-hacking [13], we focus on zero-order effects because the current cumulative knowledge in organizational research is built using these bricks [36]. Indeed, Aguinis et al. [35] report that 90% of meta-analytic summaries are in the Pearson’s *r* correlation metric and 10% are in the Cohen’s *d* metric. Beyond organizational research, meta-analyses in other fields show that standardized mean differences and Pearson’s *r* correlations are also the most commonly used inputs (e.g., [37,38]). Although it is common to use procedures such as meta-analytic structural equation modeling, these approaches ultimately start with zero-order correlations to produce meta-analytic correlation matrices upon which further analyses are performed. For this reason, we test for *p-*hacking in our cumulative knowledge based on wide arrays of meta-analytic inputs (i.e., zero-order correlations), and not the *p*-values representing effects that are rarely meta-analyzed^i^ (e.g., model fit comparisons).

In this study, we examine whether the evidence on which organizational meta-analysts rely presents with evidence of *p*-hacking and evidential value. (The latter refers to evidence that the effect is nonzero [13].) That is, we wish to describe the degree of bias present among a large sample of effect sizes that serve as an ocean of findings from which meta-analysts pluck (and have been plucking) for decades to inform cumulative knowledge. This leads us to ask our first question:

Research Question 1. *What is the prevalence of* p*-hacking and evidential value in the cumulative evidence of organizational research*?

To answer this question, we requested and obtained data from metaBUS, presently the largest database of manually curated research findings in the social sciences (i.e., more than 1,000,000 effects; [39]). Using two analytical approaches (z-curve, critical bin comparison), we extend QRP research by conducting a robust, large-scale analysis of *p*-hacking in organizational research. Further, we also attempt to explain variance in *p*-hacking attributable to publication year, journal prestige, and authorship team size (as detailed below). Our results offer important insight into our risk exposure from *p*-hacking, a meta-scientific effort that can be used to “calibrate the scientific ecosystem toward higher standards of efficiency, credibility, and quality” [40, p. 13].

The remainder of our manuscript is organized as follows. First, we review the literature on possible causes of *p*-hacking and float three additional research questions. Next, we review existing *p*-hacking investigations and approaches for its detection, highlighting their benefits and drawbacks. We then explain the methods and report the results of our study. Finally, we discuss our findings, implications for research and practice, and limitations.

### Predictors of *p*-hacking

Before the early 1990s, there was insufficient researcher behavior data and other tools to test for misconduct on a large scale [2]. The 1992 National Academies of Science report coined the phrase “QRP” in attempts to foster higher standards for research integrity [2]. Nearly 30 years later, QRPs such as *p-*hacking remain problematic and are prevalent in management and organization studies [1,41], and we seek to understand what might explain *p-*hacking. Specifically, we highlight that changing research environmental characteristics (e.g., the movement towards open science, advanced statistical software), increasing pressures to publish in high-impact outlets, and authorship team characteristics may influence researchers’ likelihood to engage in *p*-hacking.

#### Publication year

Why might the changing research climate influence the prevalence of *p-*hacking? In the last decade, open science, reproducibility [42], and replicability [43,44] have seen increased research attention. The highly cited Reproducibility Project [45] reported a 47% chance of successful replication, operationalized as effects in the same direction and similarly significant or nonsignificant, across 100 published findings in psychology. This and similar studies have led academics to increasingly question the robustness of reported evidence. Relatedly, availability of platforms like retractionwatch.org have increased our awareness of research misconduct. Still, it seems that research misconduct has not been eradicated [46,47]; indeed, retractionwatch.org includes dozens of retracted papers in organizational research over the last decade. Moreover, Olsson-Collentine et al. [48] reported that, compared with other areas of psychology, organizational psychology most frequently report “marginally significant” *p-*values (i.e., .05 < *p* ≤ .10), although this is not necessarily a QRP. Nonetheless, increased digital capabilities and the push towards open science create an opportunity for researchers to publicly share their data, tools, and methods [44], which might deter researchers from *p*-hacking. Furthermore, some journals now offer a results-blind review process option (e.g., *Journal of Business and Psychology*) [49] or the option to submit a study as a registered report, both of which are tools that could reduce author concerns of rejection based on findings’ statistical significance.

In addition, computing advances have allowed for increasingly complex and rapid statistical analyses [14,50]. However, they may also facilitate or even encourage *p*-hacking. For example, in structural equations modeling (SEM), researchers can inspect modification indices to add or remove parameters in attempts to find better-fitting models or significant paths. Blindly following modification indices might yield, for example, error terms correlated without justification. Researchers are able to iterate through many combinations of omitted variables, observations, parameters, or potential outliers until the desired result is obtained. We do not intend this as a criticism of SEM; this is just one of many possible examples of advanced statistical techniques and may apply to more simple model regression techniques as well (e.g., stepwise model simplification). Although increased automation and computing power can be advantageous (e.g., to conduct Monte-Carlo simulations), Butler et al. [1] and Crede and Harms [51] point out that users may not completely understand analytic implications, resulting in potentially erroneous analyses.

In short, over time, researchers have experienced major changes to the research environment. Thus, we ask Research Question 2a: *To what extent does publication year explain variance in* p*-hacking*?

#### Journal prestige

Manuscripts that contain statistically significant findings and/or prioritize theoretical contributions over replications or null findings are more likely to survive the review process [52–56]. Publishing in high-impact journals is a top priority for most researchers [57], thus, in response to journals’ emphasis on statistical significance and/or novelty over replication [45,58], researchers may engage in QRPs to increase manuscript acceptance likelihood [34]. Furthermore, in the presence of pressure to publish, individuals may be more inclined to engage in unethical behavior for career advancement [59]. As noted by Atwater et al. [60, p. 1179] “authors seek publication in high visibility journals by any means, fair, or foul”. Similarly, Butler et al.’s [1, p. 95] qualitative study of business school researchers indicate frequent “playing with numbers,” “playing with models,” and “playing with hypotheses,” which is likely caused by insufficient methodological training, pressures to publish [10], or journal or reviewer expectations.

In organizational research, higher-impact journals tend to emphasize novelty of findings published in their journals, and existing studies reveal that QRP prevalence and journal impact factor are positively related [10,61]. Thus, findings published in higher-impact journals may be less trustworthy (i.e., false positives; [57]). In fact, Butler et al. [1] provide practical recommendations to curb *p*-hacking practices such as improving graduate training, but lament that the institutional and structural pressures described above are unlikely to change [62]. This leads us to ask Research Question 2b: *To what extent does journal prestige explain variance in* p*-hacking*?

#### Authorship team size

Finally, we explore authorship team characteristics as a predictor of *p-*hacking. As an example, Baum and Bromiley [34] reported a nonlinear relationship between employer prestige and *p-*hacking: those employed by mid-ranked schools were more likely to exhibit *p-*hacking than those of lower-and top-tiered schools. In addition, females and researchers who earned degrees at highly ranked schools were less likely to *p*-hack. Furthermore, they reported positive yet nonsignificant relations with author publication record and faculty rank. However, their analyses were limited to articles with fewer than three authors. Thus, it remains unknown whether authorship team size relates to *p-*hacking.

Publishing manuscripts takes collaborative effort and decisions. Previous research has found both positive and negative effects of social influence on individuals’ ethical decision-making and behaviors [63,64]. Indeed, existing studies indicate that the presence of another person can reduce individuals’ likelihood to engage in unethical behavior [65,66]. Thus, researchers may be less likely to *p-*hack when collaborating with colleagues. Another possibility is that co-authors might be pressured to conform to questionable norms when working with others and, therefore, *more* likely to engage in *p*-hacking when working with others (cf. [63,67,68]. Considering the ethical decision-making and behavior literature, one might expect differences in *p-*hacking when working alone or in a group. Thus, we ask Research Question 2c: *To what extent does authorship team size explain variance in* p*-hacking*? (We conduct team size analyses in two ways: Using a continuous team size variable and then a dichotomous variable [i.e., solo- vs. multi-author].)

### Approaches for *p*-hacking detection

There exist several statistical techniques to test for the presence of *p*-hacking. In this section, we describe three (see Table 1 for a summary of each approach discussed, advantages, limitations, and the relevance of its limitations to the present study). Some approaches (e.g., critical bin comparison, *p-*curve) test for not only deviations from the *p*-value’s expected frequency distribution (i.e., *p-*hacking), but also the presence of evidential value.

**Table 1:** Approaches to detect *p-*hacking.

Statistical approach	Purpose of test	Source	Major advantage(s) of approach	Critique/limitation of approach	Source of critiques	Risk of criticism for present study
Critical bin comparison	Test difference of proportions of frequency of *p-*values between two specified bins; a greater proportion of *p-*values in the upper bin (just under .05) indicates evidence of *p-*hacking.	Head et al. (2015) [13]	1. Its specificity of testing for differences at various intervals along the *p-*value continuum. 2. Resistant to extreme *p-*values. 3. Appropriate for analyses of heterogenous data sets. 4. Requires very few assumptions.	Extant applications have analyzed rounded *p-*values, which creates ambiguous *p-*values (e.g., those rounded to two decimal places).	Hartgerink (2017) [81]	We calculate exact *p-*values using Pearson’s *r* and *N*.
				Requires large sample size for adequate power.	The present study	Our large database offers .80 power based on Head et al.’s mean effect *g =* .047.
*p-*Curve	Analyzes the complete distribution of statistically significant *p-*values for a set of independent findings; left-skewed *p-*curves indicates *p-*hacking and lack of evidential value.	Simonsohn et al. (2014) [73]	1. Able to assess if a set of statistically significant findings contains evidential value.	Extant applications have relied on automated text mining methods to obtain *p-*values.	Bishop & Thompson (2016) [74]	We do not use automated text mining methods; the metaBUS is a manually curated database of effect sizes, of which we calculate exact *p-*values.
				Not robust to data that violates its underlying assumptions (e.g., narrow set of findings, not a heterogenous dataset).	Bishop & Thompson (2016) [74]; Simonsohn et al. (2014) [5]	Not applicable to the present study. We do not utilize the *p-*curve analysis.
				Assumes all studies have the same population effect size.	Schimmack & Brunner (2017) [17]	
				Provides no information about publication bias because non-significant results are not shown.	Bartos & Schimmack ("Z-Curve: An even better *p-*curve")	
				Does not apply to studies analyzed using discrete test statistics (e.g., difference of proportions test); imposes a number of technical complications.	Simonsohn et al. (2014) [73], p. 545	
				Less likely to conclude data have evidential value when a covariate correlates with the independent variable of interest (e.g., in the absence of random assignment).	Simonsohn et al. (2014) [73], p. 546	
				Fail to detect studies that lack evidential value because the *p-*curve is right-skewed when an effect is real, but only mildly left-skewed when a finding is *p-*hacked. *P-*curve will not detect the latter if a set of true effect findings are combined with non-existent ones.	Simonsohn et al. (2014) [73], p. 546	
Z-Curve	Uses the full distribution of *p-*values to estimate the expected replicability rate (ERR), expected discovery rate (EDR), and observed discovery rate (ODR). The EDR and ODR indicate whether one observes more or fewer statistically significant values that one would expect.	Schimmack and Brunner (2017) [17]; Bartoš and Schimmack (2022) [18]	1. Accounts for statistical power. 2. Provides information about the presence and amount of selection bias. 3. Uses a finite mixture model to estimate two statistical parameters of the data (EDR, ERR).	Adjusts estimates for selection effects, but not for the use of QRPs. QRPs may lead to an underestimation of average power. This could be a limitation or considered as a conservative bias that is justified because researchers may be deterred to engage in QRPs if these practices lead to lower estimates.	Schimmack and Brunner (2017) [17], p. 27	The present study is at low risk because likely few of the effect sizes were directly hypothesized.

*Note*. We note that across all approaches, Lakens (2015) [83] criticizes that *p-*hacking should not be assessed using just values *p* < .05, rather, we should account for the entire distribution if *p-*values. To overcome this criticism, we utilize the z-curve.

#### The *p*-curve test for *p*-hacking

The *p*-curve [5] analyzes the complete distribution of *p*-values (i.e., 0 ≤ *p* ≤ 1) drawn from independent studies of a given effect. Analyses of *p*-curves have appeared in a variety of disciplines (e.g., [20,23–26,69–72]). In this analytic approach, right-skew indicates the presence of true effects (i.e., evidential value) and a bump in the curve just under .05 indicates *p*-hacking.

Applications of the *p*-curve analysis are limited in several ways. The limitations are explained in detail by Bruns and Ioannidis [15] and Simonsohn et al. [5,73]. However, the *p-*curve analysis is inappropriate for our study context primarily because it was designed to be applied to findings pertaining to a single topic (i.e., homogenous with respect to substantive relation) and assumes that all studies have the same population effect size [17]. Indeed, researchers have cautioned against using the *p-*curve on data that does not meet the underlying assumptions of the *p-*curve analysis (e.g., narrow set of findings; [74]). Additional limitations are described in Table 1. In the present study, we do not utilize the *p-*curve because our datasets are heterogeneous.

#### The z-curve test for *p-*hacking

The z-curve tests for *p-*hacking by estimating the expected distribution of significant *p-*values [17,18]. First, it presents the observed discovery rate (ODR), which is the observed proportion of statistically significant results in a set of studies. Second, it presents the expected discovery rate (EDR), or “the mean power before selection for significance; in other words, the mean power of all conducted values with statistically significant and non-significant results” [18, p. 3]. Stated differently, the ODR and EDR indicate whether we observe more or fewer statistically significant values than expected. Finally, it provides the expected replicability rate (ERR), or the predicted success rate of exact replication studies [18]. The z-curve is a relatively nascent approach and we aim to apply it in organizational research as an analytical approach for *p-*hacking detection.

The z-curve is advantageous primarily for two reasons. First, it considers the entire distribution of *p-*values to estimate two statistical parameters of the data (EDR, ERR, for additional detail, see [17,18]). Second, it accounts for the power of the sample size. That is, it allows for heterogeneity in power by assuming that different samples have different means. This is the primary advantage over the *p-*curve, which assumes equal power. With heterogenous datasets like ours, the z-curve fits the assumptions of varying power across samples.

The z-curve is not without limitations. First, it assumes that all studies used the same criterion for statistical significance (alpha = .05). However, Schimmack and Brunner [17] note that this is a minor problem considering the overwhelming majority of studies do indeed utilize the *p* < .05 criterion as the benchmark to assess statistical significance of an effect, and can be explored further by conducting z-curve estimations at different alpha criteria. Indeed, this benchmark is regarded as a default in many social science fields [75], especially psychology and business–fields from which metaBUS draws. Second, the z-curve does not adjust parameter estimates for the use of QRPs, which may lead to an underestimation of average power. This could be considered as either a limitation or a justifiable conservative bias because researchers may be deterred to engage in QRPs if these practices lead to lower estimates [17]. The z-curve is one of two analytic approaches used in the present study.

#### The critical bin comparison analysis for *p*-hacking

Head et al.’s [13] approach tests for deviations from the *p*-value’s expected frequency distribution. It tests for both the presence of evidential value and *p-*hacking by comparing the frequencies of *p-*values in critical bins. A right-skewed *p*-value distribution indicates evidential value (i.e., presence of a true nonzero effect), where one would expect an increasing frequency of smaller *p*-values, or a greater frequency of values in the lower bin (.000 ≤ *p* < .025) compared to the upper bin (.025 ≤ *p* < .050). In contrast, in the presence of a null effect, one would expect a uniform (i.e., flat) distribution of *p*-values. Importantly, given a sufficiently large sample, *p*-value distributions that are neither uniform nor right-skewed are unexpected. When such phenomena occur, researchers may be at increased risk of making false inferences regarding the true association of variables.

To test for *p-*hacking, this analysis compares the frequencies of *p*-values in two different critical bins: .040 ≤ *p* < .045 (i.e., the lower bin) and .045 ≤ *p* < .050 (i.e., the upper bin). Upper-bin frequencies (i.e., the “just barely significant” bin) should not be significantly greater beyond chance levels unless, as an example, researchers are engaging in QRPs to achieve the conventional *p* < .05 level.

An advantage of the critical bin comparison approach is its specificity of testing for differences at various intervals along the continuum. Indeed, the test is resistant to extreme *p*-values and, thus, essentially immune to outliers. Moreover, it is suitable for analyses of heterogenous data sets and has been applied in the areas of management [34], psychology [32,33], orthopedics [27], and biology [76]. It is worth noting that Head et al.’s [13] approach requires very few assumptions. Indeed, it is possible that a researcher *p*-hacks a finding of *p* = .051 resulting in *p* = .039. In cases like these, the Head et al. approach would not include the finding in either of the critical bins because the cutoff for the lower bin is .040. We view this not as a limitation, but a strength of the approach–that is, it makes very few assumptions. Still, existing applications of critical bin comparisons (e.g., [13]) present with two perennial limitations that we seek to overcome in the present study: inadequate statistical power and relation type heterogeneity. We describe each in turn.

Unfortunately, researchers have paid little attention to statistical power in primary studies [77], and the same might be said of QRP research or meta-science research [78]. Indeed, the Head et al. [13] approach is not immune to statistical power concerns. Rather, it requires one to begin with exceptionally large samples of existing *p*-values (i.e., findings) because *most* findings do not fall within the range .04-.05 which, in a uniform distribution, would represent a mere 1% of all findings. More findings, yet still a small minority, would exist in the two bins in the presence of evidential value.

Imagine a researcher were to conduct a difference of proportions test according to Head et al. [13] on a given meta-analytic data set. Imagine further the presence of evidential value among 312 effects included in an existing meta-analysis (job satisfaction-performance; [79]), a relatively large data set by modern standards. In this dataset, presented in Judge et al.’s [79] Appendix, 10 of the 312 (i.e., 3.2%) of the correlations’ *p*-values exist in the range .040 ≤ *p* < .050. The lower bin contains seven *p*-values and the upper bin contains three. To express this proportion as an effect size, we rely on *g* (Cohen, 1988), which refers to deviation from probability = .50. Following the present example, 30% upper bin membership resolves to *g* = -.20 because it is .20 less than .50. Is sufficient statistical power present to detect a difference in these proportions? No. In fact, the observed power is .15 according to a one-tailed binomial exact proportion sign test using G*Power 3.1.9.7 [80]. (Note that Judge et al.’s meta-analysis is one of the largest reported to date in organizational research on a single bivariate relation).

So, how many *p*-values must be present across the two bins to achieve sufficient statistical power? The answer would depend on the expected effect size. An analysis based on Head et al.’s [13] findings reveal a mean sample size-weighted effect of *g* = .047 (i.e., 54.7% of *p*-values in the upper bin; see Table 2). Using G*Power [80], we conducted a one-tailed binomial exact proportion sign test power analysis to have an informed estimate of the necessary sample size needed for our critical bin comparisons to achieve .80 power. This analysis revealed that 705 observations would be needed in the range .040 ≤ *p* < .050 to detect an effect of *g* = .047 with .80 power. However, Head et al.’s [13] findings rarely reached this level of power. Indeed, our post-hoc analyses reveal that Head et al.’s [13] power averaged .435 and only three of their 14 data sets (i.e., 21%) achieved .80 power (see Table 2).

**Table 2:** Post-hoc power analyses of Head et al. (2015).

Research field	Effect size (*g*)	*N*	Post hoc power
Agricultural and veterinary sciences	.115	26	.277
Biological sciences	.047	773	.835
Chemical sciences	.048	31	.102
Earth sciences	.500	4	N/A
Education	-.029	17	.042
Engineering	-.071	28	.170
Environmental	.155	29	.431
Information and computing sciences	.100	50	.336
Mathematical sciences	-.500	3	N/A
Medical and Health sciences	.047	3,262	1.000
Multidisciplinary	.040	1,388	.906
Psychology and cognitive sciences	.133	79	.722
Studies in human society	-.227	11	.387
Technology	.000	6	.016

*Note*. Power estimated using the one-tailed binomial exact proportion sign test using G*Power 3.1.9.7. N/A indicates G*Power was unable to estimate.

However, consider the implication: If 705 *p*-values between .040 ≤ *p* < .050 are needed to conduct Head et al.’s [13] analysis with sufficient power, and only 3.2% of findings exist in the range .040 ≤ *p* < .050 (as was the case for Judge et al. [79]), one would require a starting database of roughly 22,000 *p*-values, a size rarely achieved in existing *p*-hacking research.

Although a second advantage of the Head et al. [13] approach is its insensitivity to finding type heterogeneity, it would nonetheless be valuable to account for variance attributable to finding type. Head et al. [13] extracted all *p*-values from articles (e.g., using document extraction algorithms that identify character strings such as “*p* =,” “*p* <,” and “*p* >”). Although this approach offers the requisite large volume of *p*-values, it does so at the expense of control for the type of finding under investigation and, often, the exact *p*-value [74]. Thus, phrases such as *the focal relation between x and y was not moderated by participant age*, p *=* .*05* or *the manipulation check revealed that participants perceived x as less than y* (p *=* .*04*) would have been extracted for inclusion in such analyses despite their relatively incidental nature. (To our knowledge, findings like these are rarely summarized using meta-analysis.) Moreover, as noted by Hartgerink [81], rounding the *p-*value to two decimal places is a limitation of the existing applications of critical bin comparisons.

### The present study

In the present study, we seek to overcome existing limitations of *p-*hacking detection by conducting large-scale analyses that account for bivariate relation type and, when possible, provide sufficient statistical power. We obtained data from the metaBUS team, in which each effect size is manually classified according to a taxonomy containing roughly 5,000 variables and constructs studied in the scientific space. The metaBUS taxonomy starts with broad branches (e.g., behaviors, attitudes), which branch into finer levels (e.g., behaviors → performance, behaviors → turnover). Importantly, because effect sizes vary as a function of bivariate relation type [82], *p*-values should follow suit assuming their samples sizes do not differ substantially.

We also sought to maximize statistical power. As described earlier, for the critical bin comparisons, our a priori power analysis led us to seek data sets with at least 705 *p*-values in the range .040 ≤ *p* < .050. For this reason, we chose not to nuance our analyses by specific bivariate relations (e.g., performance-turnover). Further, some researchers have criticized methods to detect *p-*hacking not only for failing to consider power, but also failing to consider the *p* ≥ .05 range [83]. To address these concerns, we conduct a supplemental analysis using the z-curve which addresses both of these limitations. This serves as another lens to interpret our results more robustly.

## Materials and methods

### Transparency and openness

All analyses were conducted using R version 4.2.1. The R code and dataset is provided at https://osf.io/qaj3m/.

### Dataset

The first author contacted a member of the metaBUS team to request access to the metaBUS database (version 2018.09.09), which contains 1,038,238 correlation coefficients from 26 journals published between 1980–2017 [39]. Requested were all rows of the following database fields: correlation coefficient, sample size, journal source, publication year, digital object identifier (DOI), and the taxonomic classification of bivariate relation type. We calculated *p-*values for each correlation coefficient using the reported sample size. (In organizational research, most correlations are reported to two or three decimal places.) In doing so, this reduces concern of relying on rounded *p-*values, or *p-*values collected via automated text mining methods [74,81]. For correlations whose variable pairs presented with different sample sizes, we relied on the lesser of the two.

### Database subsetting by bivariate relation type

We subsetted the complete metaBUS database based on bivariate relation type. To obtain adequate statistical power, we identified the five most frequent construct types (i.e., higher-order taxonomic categories) studied in organizational research [82] and their corresponding five-digit taxonomic identifiers in the metaBUS platform (i.e., attitudes [20115], behaviors [20203], organizational characteristics [20521], psychological characteristics [30085], and objective individual characteristics [30088]). When crossed, the five construct types give rise to 15 finding type pairs (see Table 4).

**Fig 1:**
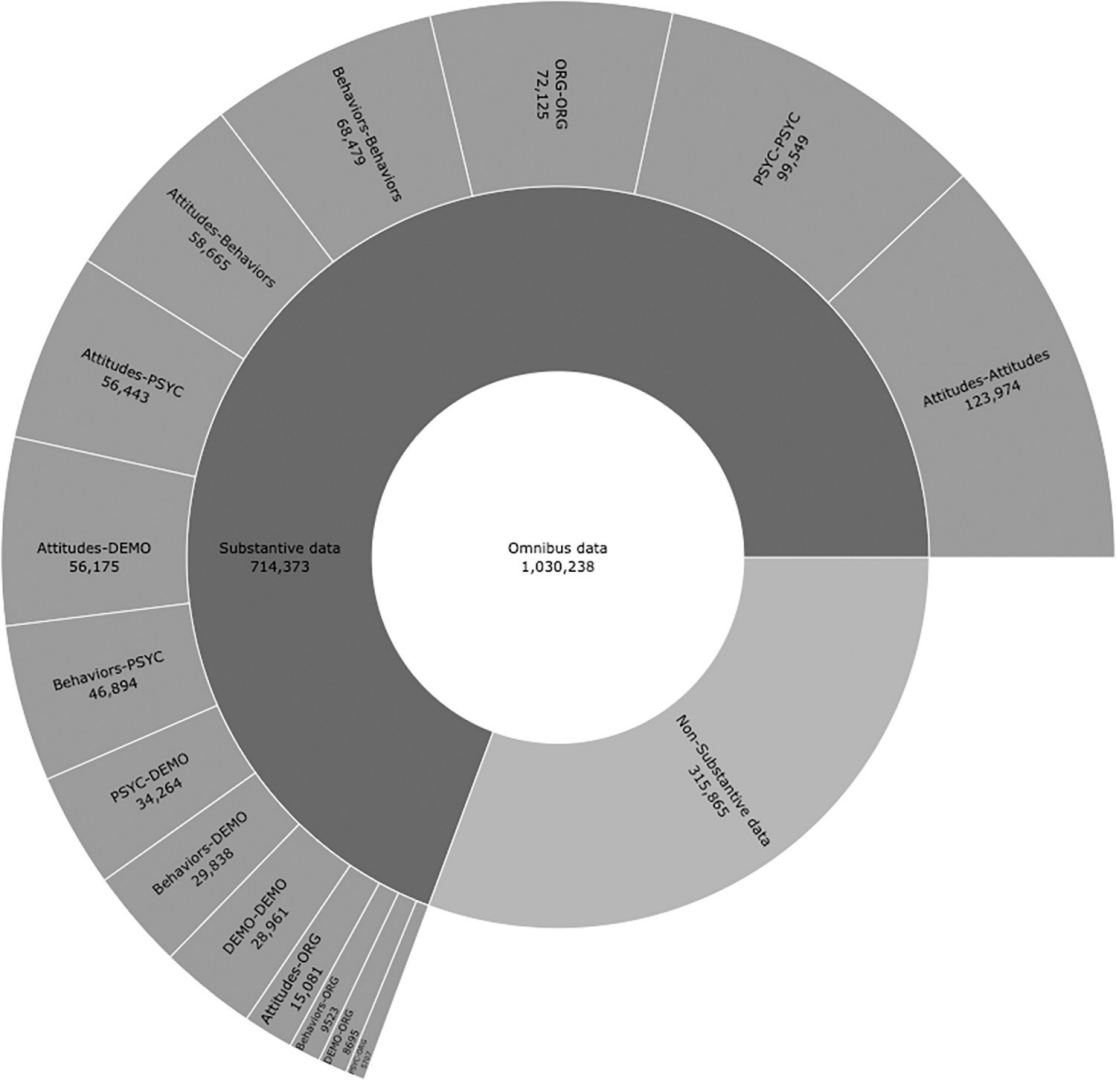
Breakdown of metaBUS dataset. *Note*. PSYC = Psychological characteristics; DEMO = Objective person characteristics / demographics; ORG = Organizational characteristics. The three smallest regions read (from left to right): Behaviors-ORG (*N* = 9,523), DEMO-ORG (*N* = 8,695), and PSYC-ORG (*N* = 5,707).

To provide additional lenses through which to view our results, next we collapsed across the above 15 bivariate relation types to create an aggregate *substantive* database. As shown in Fig 1, the substantive dataset accounts for 68.8% of the complete metaBUS dataset. Lastly, we group the data that do not fall into one of the 15 bivariate relation types into a *non-substantive* dataset. We note that not all that we call “(non)substantive” are certainly (non)substantive, however, we chose this approach to offer results views where hypothesis relevance likelihood is higher or lower (e.g., psychological characteristics-behavior relations are likely to be hypothesis-relevant). The entire breakdown and number of *p-*values is shown in Fig 1 and descriptives for each of these datasets (with the exception of the 15 bivariate relation type subsets; R material provided online) are provided in Table 3. Finally, for the critical bin comparisons, we created subsets limited to *p* < .05; descriptive statistics for each dataset are shown in Table 3.

**Table 3:** Descriptive statistics for data subsets.

		Authorship team size			2021 SJR			Publication year		
	*N*	M (*SD*)	Median	[Min, Max]	M *(SD*)	Median	[Min, Max]	M (*SD*)	Median	[Min, Max]
Complete metaBUS										
All *p*-values	1,038,238	2.85 (*1*.*37*)	3	[1, 34]	4.18 (*2*.*98*)	3.01	[.57, 10.87]	2006 (*7*.*99*)	2007	[1980, 2017]
Only *p <* .05	581,285	2.88 (*1*.*40*)	3	[1, 34]	4.04 (*2*.*90*)	2.90	[.57, 10.87]	2006 (*8*.*07*)	2007	[1980, 2017]
Substantive										
All *p*-values	714,373	2.87 (*1*.*40*)	3	[1, 34]	4.02 (*2*.*90*)	2.90	[.57, 10.87]	2006 (*7*.*94*)	2007	[1980, 2017]
Only *p <* .05	423,952	2.90 (*1*.*42*)	3	[1, 34]	3.89 (*2*.*81*)	2.90	[.57, 10.87]	2006 (*7*.*96*)	2007	[1980, 2017]
Non-substantive										
All *p*-values	315,865	2.81 (*1*.*31*)	3	[1,24]	4.52 (*3*.*12*)	3.09	[.57, 10.87]	2006 (*8*.*12*)	2007	[1980, 2017]
Only *p <* .05	157,333	2.81 (*1*.*32*)	3	[1,24]	4.45 (*3*.*09*)	3.09	[.57, 10.87]	2005 (*8*.*33*)	2007	[1980, 2017]

*Note*. M = Mean; *SD* = Standard deviation; Min = Minimum; Max = Maximum. SJR = SCImago journal rank.

### Data coding

#### Dependent variable

For critical bin comparisons [13], a dummy variable was coded as 1 if the *p*-value was in the upper bin (.045 ≤ *p* < .050) or 0 if the *p*-value was in the lower bin (.040 ≤ *p* < .045) and *p*-values outside this range were excluded. For the z-curve, the complete distribution (i.e., .000 ≤ *p* < 1.000) was used and no binning was applied.

#### Journal prestige

We relied on the impact factors from 2021 *SCImago Journal* as an indicator of journal prestige [84]. The data were retrieved in December 2022.

#### Authorship team size

We collected authorship team size for each article. To this end, we extracted the full reference from each source by submitting its DOI value to the cr_cn() function in the R package rcrossref [85]. Using this extracted information, we used automation to count the number of authors in each reference.

### Analytical approach

#### Research question 1

Our first research question addresses the frequency of *p*-hacking. We employed two analytical strategies. First, we ran the z-curve on each of the datasets using the zCurve R package [86]. We specified an expectation-maximization model with 5,000 bootstrap samples. Second, similar to previous *p-*hacking studies, we employed a series of critical bin comparisons that compared frequencies of *p-*values in the upper (.045 ≤ *p* < .050) and lower (.040 ≤ *p* < .045) bins for each of the data sets. Next, we tested for the presence of evidential value (i.e., whether *p-*values between .000 ≤ *p* < .025 were more frequent than between .025 ≤ *p* < .050) in each of these datasets. For bin frequency comparisons, we relied on the binom.test function of R base [87].

#### Research question 2

To test our second research question, we again apply two analytic approaches, one set based on the z-curve and another based on critical bin comparisons.

To test for predictors of *p-*hacking using the z-curve, we split the data into categories (i.e., subsets). For example, to test the effect of publication year, we split data into before and after the year 2000 (i.e., 1980–1999 vs. 2000–2017) and estimated effect sizes for each group. For authorship team size and journal prestige, we split the databases at each predictor’s median (see Table 3 for medians).

To test the effects of the predictors using critical bin comparisons, we followed Baum and Bromiley [34] and conducted a series of multilevel logit regression analyses on each dataset. We tested for publication year, journal prestige, and authorship team size as predictors of critical bin membership (i.e., 1 = upper bin; 0 = lower bin). For these analyses, we relied on the glmer function in the lme4 R package [88], which allows us to account for the non-independence of effects that arise from article level characteristics. Pseudo-*R*^2^ was calculated according to Johnson [89] using the r.squaredGLMM function of the MuMIn R package [90]. For these analyses, marginal *R*^2^ is an estimate of the variance attributable to fixed factors (i.e., model predictors) and conditional *R*^2^ is an estimate of variance attributable to fixed and random (i.e., nesting) factors combined.

## Results

### Overview

We organize our results according to our two general research questions. First, we examine the prevalence of *p-*hacking. We report Cohen’s *h* for z-curve effect sizes and Cohen’s *g* for bin comparisons. Although both effect size indices compare proportions, Cohen’s *g* is purposed specifically for the case where the expected proportion is .50 (i.e., critical bin comparisons). Second, we examine the influence of our three predictors of interest.

### Research Question 1. *P*-hacking Prevalence

#### Z-curve estimation

**Fig 2:**
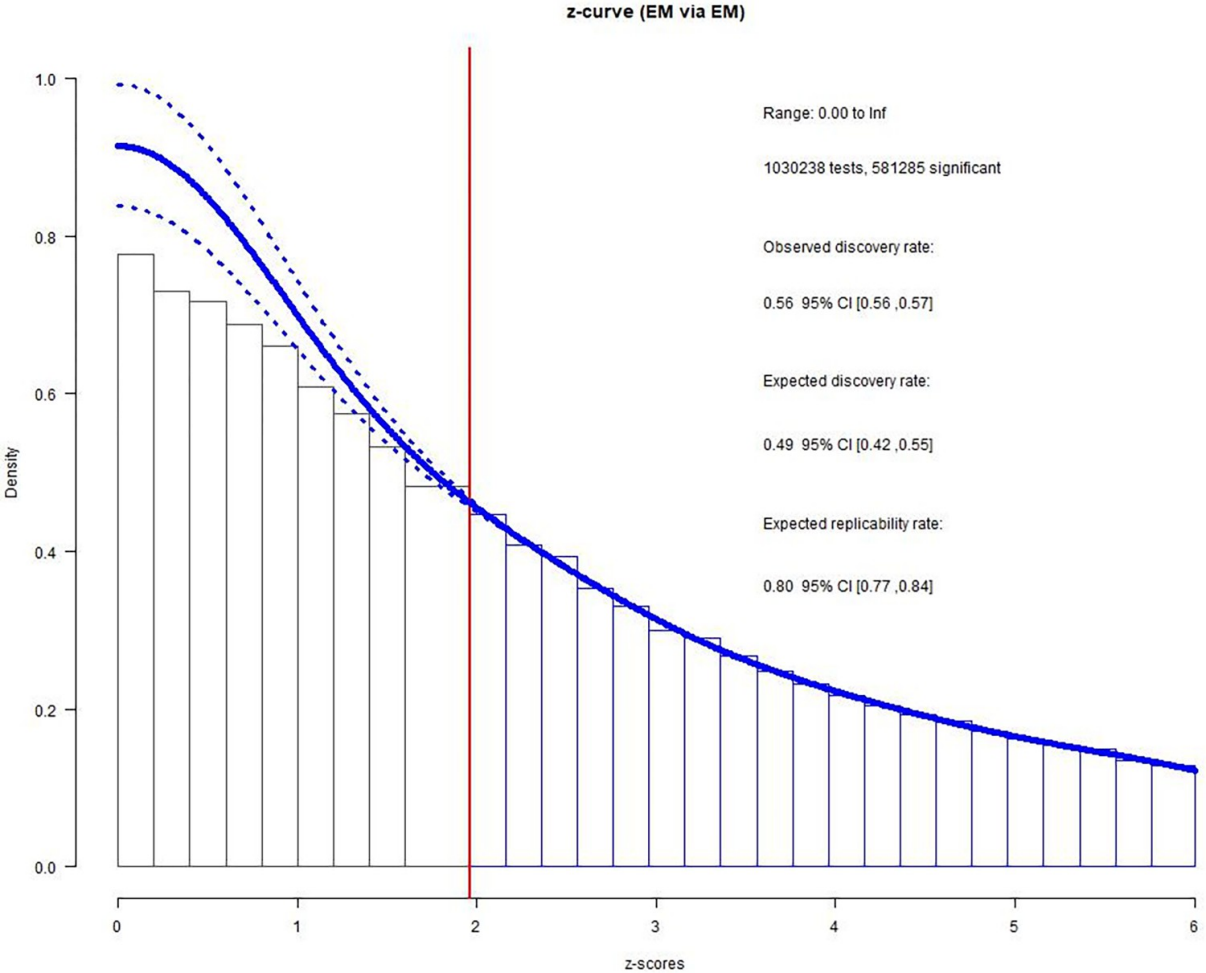
Z-curve results for the complete metaBUS dataset.

As shown in Fig 2, the z-curve for the complete dataset indicated significantly more observed (ODR = .564, 95% CI [.563, .565]) than expected (EDR = .489, 95% CI [.424, .555]) significant findings (*h* = .151).

For the 15 bivariate relation types, we observed nine subsets in which the observed estimate was greater than the expected estimate (i.e., nine positive effects; six negative effects), none of which reached statistical significance. (A graph of each bivariate relation type’s results are included in S1 Fig of the online supporting information; see Table 4 for a summary). Across the 15 bivariate relation types, *h* ranged from -.160 to .210 (Mean = .019, *SD* = .097).

**Table 4:** *P-*hacking results according to the z-curve approach.

	Total number of tests	Significant number of tests	ODR (95% CI)	EDR (95% CI)	ERR (95% CI)	*h*
Complete metaBUS dataset	1,030,238	581,285	.56 (.56, .57)	.49 (.42, .55)	.80 (.77, .84)	.15*
1980–1999	229,917	131,209	.57 (.57, .57)	.59 (.50, .67)	.80 (.77, .83)	-.04
2000–2017	800,321	450,076	.56 (.56, .56)	.48 (.41, .55)	.81 (.77, .84)	.17*
Authorship team size (low)	427,068	238,295	.56 (.56, .56)	.50 (.42, .57)	.80 (.77, .83)	.11
Authorship team size (high)	522,542	298,562	.57 (.57, .57)	.52 (.44, .59)	.81 (.78, .84)	.11
Journal prestige (low)	505,077	293,832	.58 (.58, .58)	.55 (.48, .63)	.81 (.78, .84)	.06
Journal prestige (high)	524,067	286,736	.55 (.55, .55)	.48 (.41, .55)	.80 (.76, .83)	.13
Non-substantive dataset	315,865	157,333	.50 (.50, .50)	.42 (.34, .49)	.76 (.73, .80)	.16*
Substantive dataset	714,373	423,952	.59 (.59, .59)	.59 (.52, .65)	.82 (.79, .85)	.01
PSYC-PSYC	99,549	70,179	.70 (.70, .71)	.66 (.56, .75)	.87 (.83, .90)	.10
Attitudes–Attitudes	123,974	94,354	.76 (.76, .76)	.75 (.64, .87)	.90 (.86, .93)	.02
ORG-ORG	72,125	36,849	.51 (.51, .51)	.50 (.42, .57)	.78 (.75, .82)	.02
Attitudes–DEMO	56,175	20,238	.36 (.36, .36)	.28 (.20, .37)	.66 (.62, .69)	.17
Attitudes–Behaviors	58,665	34,666	.59 (.59, .59)	.61 (.52, .70)	.80 (.76, .84)	-.04
Attitudes–PSYC	56,443	36,123	.64 (.64, .64)	.54 (.42, .66)	.83 (.80, .87)	.21
Behaviors–PSYC	46,894	24,560	.52 (.52, .53)	.55 (.42, .69)	.76 (.73, .80)	-.05
Behaviors–Behaviors	68,479	49,057	.72 (.71, .72)	.71 (.58, .84)	.87 (.84, .91)	.01
PSYC–DEMO	34,264	14,001	.41 (.40, .41)	.37 (.26, .50)	.70 (.66, .74)	.07
Behaviors–DEMO	29,838	11,036	.37 (.36, .38)	.38 (.25, .50)	.67 (.63, .72)	-.02
DEMO-DEMO	28,961	15,122	.52 (.52, .53)	.57 (.46, .68)	.78 (.74, .82)	-.09
Attitudes–ORG	15,081	7,615	.50 (.50, .51)	.53 (.41, .65)	.76 (.71, .80)	-.05
Behaviors–ORG	9,523	4,420	.46 (.45, .47)	.42 (.26, .68)	.76 (.71, .80)	.09
DEMO–ORG	8,695	3,204	.37 (.36, .38)	.36 (.24, .46)	.64 (.58, .69)	.01
PSYC–ORG	5,707	2,528	.44 (.43, .46)	.52 (.40, .71)	.76 (.70, .81)	-.16

*Note*. Asterisk (*) indicates significant effect size based on nonoverlapping 95% confidence intervals before rounding (*h*); ODR = Observed discovery rate; EDR = Expected discovery rate; ERR = Expected replicability rate; CI = Confidence interval; PSYC = Psychological characteristics; DEMO = Objective person characteristics / demographics; ORG = Organizational characteristics. Z-curve analyses were conducted using expectation-maximization with 5,000 bootstrap samples.

**Fig 3:**
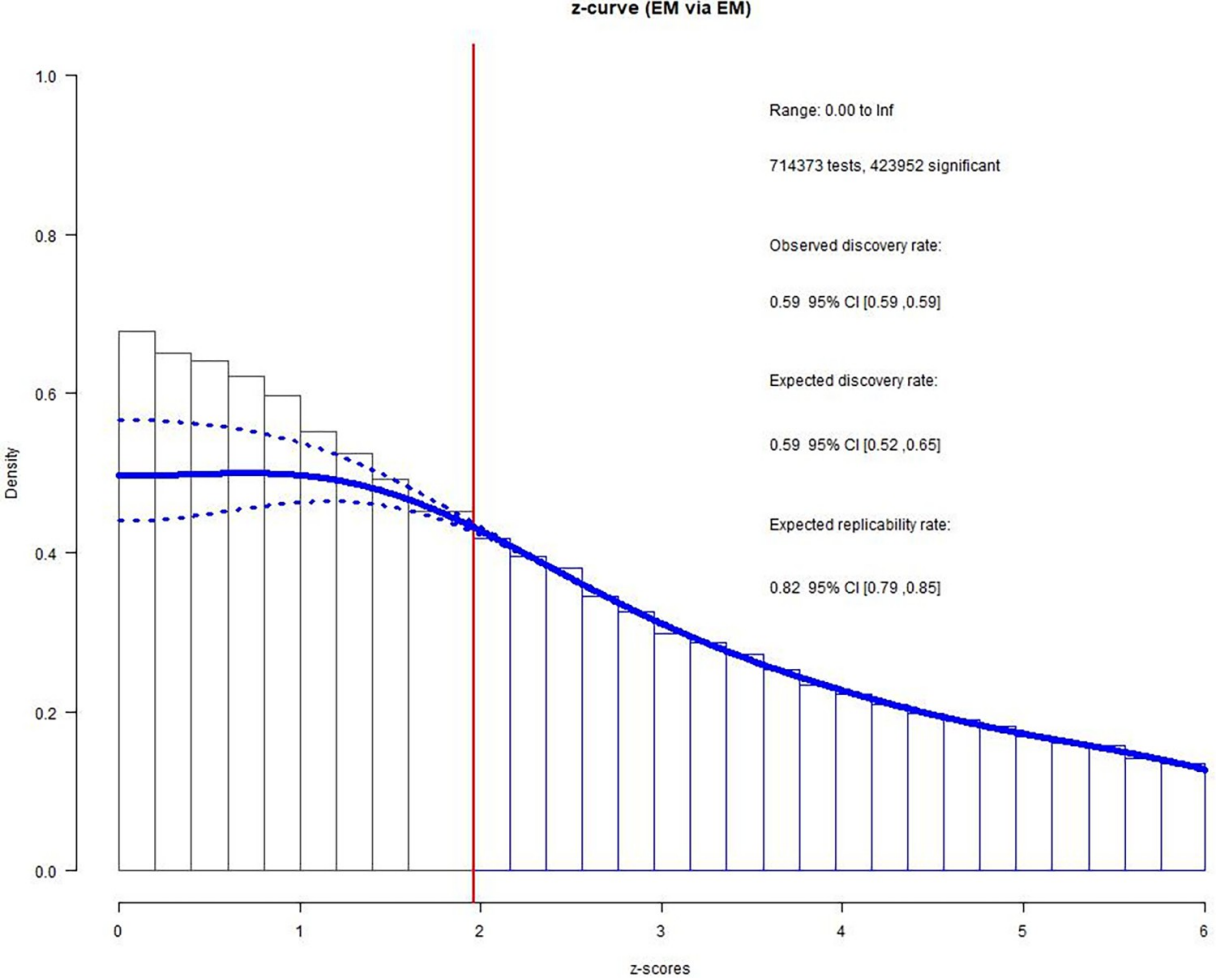
Z-curve results for the substantive dataset.

**Fig 4:**
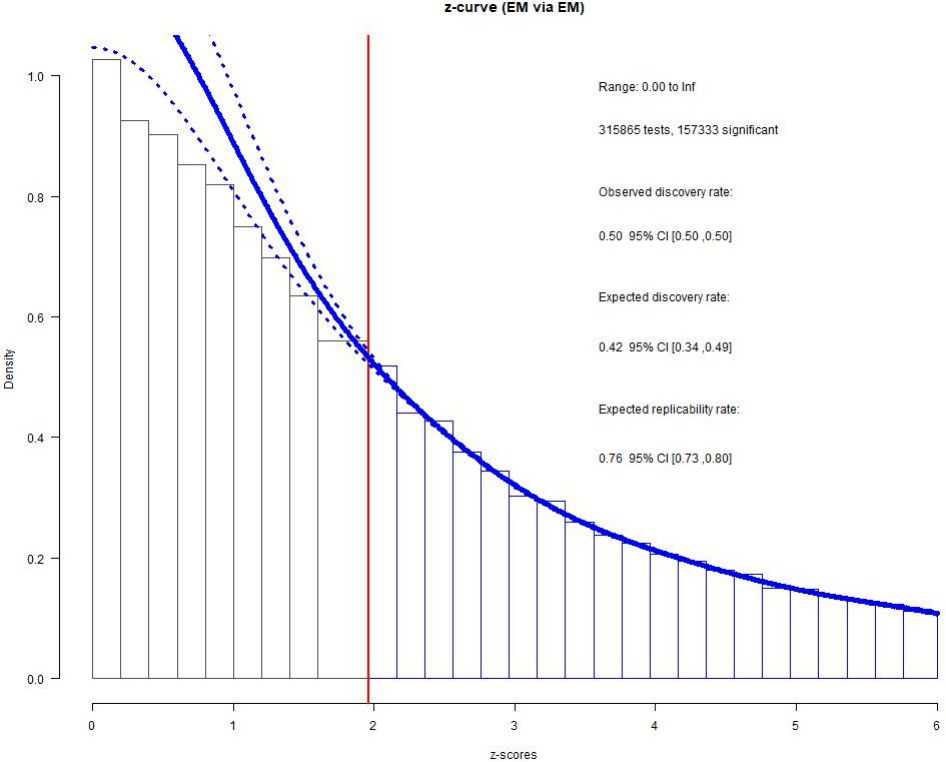
Z-curve results for the non-substantive dataset.

With the 15 bivariate relations aggregated (i.e., substantive dataset), we found no difference between observed (ODR = .593, 95% CI [.592, .595]) and expected (EDR = .587, 95% CI [.517, .654]) frequencies (*h* = .014, see Fig 3). For the non-substantive dataset, however, the z-curve revealed evidence of *p*-hacking (ODR = .498, 95% CI [.496 .500]; EDR = .417, 95% CI [.345, .490], *h* = .163), see Fig 4).

#### Critical bin comparisons

Next, following the Head et al. [13] approach, we conducted critical bin comparisons of *p*-value frequencies (i.e., .040 ≤ *p* < .045 vs. .045 ≤ *p* < .050). As shown in Table 5, the overall sample-size weighted effect size for the complete dataset failed to reach statistical significance (*g* = -.005, 95% lower confidence limit (CL) = -.012, *p* = .906). We remind readers that a negative effect size *g* indicates no evidence for *p-*hacking (i.e., a higher proportion of *p-*values in the lower bin).

**Table 5:** *P-*hacking results according to critical bin comparisons approach.

	.045 ≤ *p* < .050	.040 ≤ *p* < .045	*N*	*g*	*p*-value	Lower 95% CL	Observed power
Complete metaBUS database	7,597	7,759	15,356	-.005	.906	-0.012	0.339
Non-substantive database	2,672	2,386	5,058	.028	1.000	-0.025	0.050
Substantive database	5,211	5,087	10,298	.006	.113	-0.002	0.334
PSYC-PSYC	622	679	1,301	-.022	.946	-0.045	0.470
Attitudes–Attitudes	679	631	1,310	.018	.097	-0.005	0.351
ORG-ORG	514	504	1,018	.005	.389	-0.021	0.090
Attitudes–DEMO	534	528	1,062	.003	.439	-0.023	0.458
Attitudes–Behaviors	533	466	999	.033*	.019	0.007	0.671
Attitudes–PSYC	447	430	877	.001	.295	-0.019	0.052
Behaviors–PSYC	394	439	833	-.027	.945	-0.056	0.459
Behaviors–Behaviors	410	361	771	.032*	.042	0.002	0.548
PSYC–DEMO	310	278	588	.027	.101	-0.008	0.352
Behaviors–DEMO	258	251	509	.007	.395	-0.031	0.086
DEMO-DEMO	223	209	432	.016	.266	-0.024	0.154
Attitudes–ORG	101	124	225	-.051	.945	-0.107	0.420
Behaviors–ORG	77	85	162	-.025	.760	-0.091	0.155
DEMO–ORG	84	60	144	.083*	.027	0.011	0.600
PSYC–ORG	25	42	67	-.127	.987	-0.225	0.652

*Note*. CL = Confidence limit; PSYC = Psychological characteristics; DEMO = Objective person characteristics / demographics; ORG = Organizational characteristics.

*p < .05.

Regarding the 15 bivariate relation data sets, although we did not reach .80 power in all cases, we did achieve sufficient power, assuming *g* = .047, in eight of our 15 bivariate relations types, which compares favorably with earlier work. Across the 15 bivariate relations’ data sets, *g* effect sizes ranged from -.127 to .083 (Mean = -.001, *SD* = .047), and the relation between sample size and *g* (i.e., *p*-hacking magnitude) was *r =* .203. Ten of the effects were negative and five were positive. All effects that reached statistical significance were positive and small.

With all 15 bivariate relation type data sets combined (i.e., substantive dataset), 51% of the *p*-values existed in the upper bin, an effect that failed to reach statistical significance (*g* = .006, 95% lower CL = -.002, *p =* .113). Regarding the non-substantive dataset, the mean sample-size weighted effect size was *g* = .028 (95% lower CL = -.025, *p* = 1.000), which failed to reach statistical significance.

Finally, we tested for the presence of evidential value using critical bin comparisons [13]. As noted earlier, this analysis splits the *p* < .05 range into two equal bins and asks whether the lower bin contains a greater proportion of *p*-values. As seen in Table 6, we observed evidence of evidential value in the complete metaBUS dataset (*g* = -.422, 95% lower CL = -.422, *p* = .000), within each of the 15 bivariate relation types, with small to medium effect sizes ranging from-.351 to -.457 (Mean = -.405, *SD* = .033), as well as collapsed across all bivariate relation types (i.e., substantive dataset; *g* = -.428, 95% lower CL = -.428, *p* = .000) and, to a lesser degree, in the non-substantive dataset (*g* = -.408, 95% lower CL = -.402, *p* = .000).

**Table 6:** Evidential value results according to critical bin comparisons approach.

	.025 ≤ *p* < .050	.000 ≤ *p* < .025	*N*	*g*	*p*-value	Upper 95% CL	Observed power
Complete metaBUS dataset	45,210	536,075	581,285	-.422	0	-0.422	1
Non-substantive dataset	14,550	142,783	157,333	-.408	0	-0.402	1
Substantive dataset	30,660	393,292	423,952	-.428	0	-0.428	1
PSYC-PSYC	3,798	66,381	70,179	-.446	0	-0.444	1
Attitudes–Attitudes	4,057	90,297	94,354	-.457	0	-0.456	1
ORG-ORG	3,021	33,828	36,849	-.418	0	-0.416	1
Attitudes–DEMO	3,009	17,229	20,238	-.351	0	-0.347	1
Attitudes–Behaviors	2,946	31,720	34,666	-.415	0	-0.413	1
Attitudes–PSYC	2,619	33,504	36,123	-.427	0	-0.425	1
Behaviors–PSYC	2,484	22,076	24,560	-.399	0	-0.396	1
Behaviors–Behaviors	2,357	46,690	49,057	-.452	0	-0.450	1
PSYC–DEMO	1,682	12,319	14,001	-.380	0	-0.375	1
Behaviors–DEMO	1,519	9,517	11,036	-.362	0	-0.357	1
DEMO-DEMO	1,279	13,843	15,122	-.415	0	-0.412	1
Attitudes–ORG	702	6,913	7,615	-.408	0	-0.402	1
Behaviors–ORG	464	3,956	4,420	-.395	0	-0.387	1
DEMO–ORG	459	2,745	3,204	-.357	0	-0.346	1
PSYC–ORG	254	2,274	2,528	-.400	0	-0.389	1

*Note*. CL = Confidence limit; PSYC = Psychological characteristics; DEMO = Objective person characteristics / demographics; ORG = Organizational characteristics.

### Research Question 2. Predictors of *p*-hacking

#### Z-Curve approach

As shown in Table 4, the z-curve analysis revealed stronger evidence for *p*-hacking between the years 2000–2017 (*h* = .165, *p* < .05) than 1980–1999 (*h* = -.036). For smaller versus larger authorship team sizes, we observed a similar, positive magnitude of *p-*hacking in higher authorship team sizes (*h* = .110) and lower authorship team sizes (*h* = .105), both of which were nonsignificant. Finally, we found stronger evidence of *p-*hacking in prestigious journals (*h* = .131) than lower ranked journals (*h* = .059), both of which were nonsignificant.

#### Critical bin comparisons approach

Next, we conducted a multilevel logit regression with critical bin membership (i.e., upper vs. lower *p*-value bin) as the dependent variable regressed on three predictors (i.e., publication year, journal prestige, authorship team size). *P*-values were nested by article. The model for the complete metaBUS dataset, controlling for bivariate relation type, explained less than 1% of the variance (i.e., marginal *R*^2^ = .006), but the odds ratios for publication year (*OR =* 0.62, 95% CI [-.53, .89], *p* = .0004) and journal prestige (*OR* = 1.36, 95% CI [1.04, 1.77], *p* = .025) were significant (see Table 7). Thus, increases in publication year were associated with 0.62 multiple lower likelihood of landing in the upper bin (i.e., decrease of *p-*hacking over time), but as journal prestige increased, there was a 1.36 multiple likelihood of landing in the upper bin (i.e., more *p-*hacking in prestigious journals). Finally, authorship team size failed to significantly predict upper bin membership (*OR =* 1.08, 95% CI [.83, 1.39], *p =* .572). As an ancillary analysis, we conducted analyses to contrast solo- versus multi-author papers. When run dichotomously, the analyses yielded a non-significant odds ratio less than 1.0 (*OR* = 0.89, 95% CI [.73, 1.10], *p* = .284).

**Table 7:** Multilevel logit regression results for predictors of bin membership (complete metaBUS dataset).

	Bin membership		
*Predictors*	*Odds ratios*	*95% CI*	*p*
(Intercept)	0.69	0.53–0.89	0.004
Publication year	0.62	0.47–0.81	<0.001
Journal prestige	1.36	1.04–1.77	0.025
Authorship team size	1.08	0.83–1.39	0.572
*Controls*				
Attitudes-Behaviors	0.98	0.86–1.11	0.724
Attitudes-DEMO	0.98	0.87–1.11	0.795
Attitudes-ORG	1.06	0.95–1.18	0.281
Attitudes-PSYC	0.96	0.84–1.09	0.527
Behaviors-Behaviors	1.06	0.90–1.24	0.490
Behaviors-DEMO	1.00	0.89–1.12	0.975
Behaviors-ORG	0.99	0.89–1.10	0.845
Behaviors-PSYC	1.01	0.89–1.16	0.830
Non-Substantive Relations	0.93	0.75–1.14	0.467
DEMO-DEMO	1.06	0.93–1.20	0.390
DEMO-ORG	1.05	0.95–1.17	0.315
ORG-ORG	0.93	0.78–1.11	0.410
PSYC-DEMO	1.05	0.93–1.19	0.449
PSYC-ORG	0.98	0.88–1.10	0.781
PSYC-PSYC	0.94	0.78–1.13	0.499
*Random effects*			
σ^2^	3.29		
Τ_00_ Article	32.58		
ICC	0.91		
*N Article*	5,472		
Observations (*N*)	14,111		
Marginal R^2^ / Conditional R^2^	0.010 / 0.909		

Note. Bin membership = 1 (.045 ≤ *p* < .050) or 0 (.040 ≤ *p* < .045); CI = Confidence interval; ICC = Intraclass correlation coefficient; σ2 = Residual variance; τ00 = Variance due to nesting (i.e., article characteristics). PSYC = Psychological characteristics; DEMO = Objective person characteristics / demographics; ORG = Organizational characteristics. Marginal R^2^ is calculated according to Johnson (2014) and indexes the variance associated with fixed factors; conditional is variance attributed to fixed and random effects combined.

Next, we nuanced our results further to examine predictors of *p-*hacking for each bivariate relation type. (Each regression table is shown in S1 Table of the online supporting information; we provide overall trends here). Like the complete metaBUS dataset, publication year negatively predicted *p*-hacking in 11 of the 15 subsets (i.e., OR < 1), only one of which reached statistical significance (Attitudes-Attitudes). For journal prestige, results indicated positive relations between journal prestige and *p*-hacking in 13 of the 15 subsets, two of which reached statistical significance (Behaviors-DEMO; DEMO-DEMO). For authorship team size, we observed nine odds ratios greater than one (i.e., positive effect) and six less than one (i.e., negative effect); one of the positive effects reached significance. We ran the analyses a second time with authorship recoded into solo- versus multi-author; this analysis revealed one positive significant and one negative significant slope in two of the 15 bivariate relation types (ORG-ORG, DEMO-ORG).

Collapsed across the bivariate relation types, we conducted a similar regression analysis for the substantive dataset (see Table 8). Odds ratios for publication year (*OR* = 0.74, 95% CI [.58, .93], *p* = .010) and journal prestige (*OR* = 1.27, 95% CI [1.00, 1.60], *p* = .046) were significant. Finally, in line with the complete metaBUS dataset and bivariate relation type trends, authorship team size failed to reach statistical significance (*OR =* 1.07, 95% CI [.84, 1.35], *p =* .595). With authorship recoded into solo- versus multi-author, the analyses yielded a non-significant odds ratio (*OR* = 0.87, 95% CI [.70, 1.09], *p* = .237).

**Table 8:** Multilevel logit regression results for predictors of bin membership (substantive database; 15 relation types combined).

	Bin membership		
*Predictors*	*Odds ratios*	*95% CI*	*p*
(Intercept)	0.93	0.74 – 1.16	0.514
Journal prestige	1.27	1.00 – 1.60	0.049
Publication year	0.74	0.58 – 0.93	0.010
Authorship team size	1.07	0.84 – 1.35	0.595
*Random effects*			
σ^2^	3.29		
τ_00 Article_	22.65		
ICC	0.87		
*N*_Article_	3989		
Observations (*N*)	9482		
Marginal R^2^ / Conditional R^2^	0.006 / 0.874		

*Note*. Bin membership = 1 (.045 ≤ *p* < .050) or 0 (.040 ≤ *p* < .045); CI = Confidence interval. Marginal R^2^ is calculated according to Johnson (2014) and indexes the variance associated with fixed factors; conditional is variance attributed to fixed and random effects combined.

Lastly, we conducted the regression analysis for the non-substantive dataset (see Table 9). While publication year was significant in predicting upper bin membership (*OR* = 0.74, 95% CI [.55, 1], *p* = .047), journal prestige (*OR* = 1.23, 95% CI [.90, 1.67], *p* = .189) and authorship team size (*OR* = 1.21, 95% CI [.91, 1.59], *p* = .186) were not predictors of upper bin membership. Finally, authorship team size was nonsignificant. With authorship coded dichotomously, the analyses yielded a negative, non-significant odds ratio (*OR* = 0.95, 95% CI [.71, 1.27], *p* = .736).

**Table 9:** Multilevel logit regression results for predictors of bin membership (non-substantive database).

	Bin membership		
*Predictors*	*Odds ratios*	*95% CI*	*p*
(Intercept)	0.63	0.46 – 0.84	0.002
Journal prestige	1.23	0.90 – 1.67	0.189
Publication year	0.74	0.55 – 1.00	0.047
Authorship team size	1.21	0.91 – 1.59	0.186
*Random effects*			
σ^2^	3.29		
τ_00 Article_	20.51		
ICC	0.86		
*N*_Article_	2,187		
Observations (*N*)	4,629		
Marginal R^2^ / Conditional R^2^	0.006 / 0.863		

*Note*. Bin membership = 1 (.045 ≤ *p* < .050) or 0 (.040 ≤ *p* < .045); CI = Confidence interval. Marginal R^2^ is calculated according to Johnson (2014) and indexes the variance associated with fixed factors; conditional is variance attributed to fixed and random effects combined.

## Discussion

Using two analytical approaches, we observed that the extent and magnitude of *p-*hacking in the corpus of organizational research findings used as input to meta-analyses exists but is relatively small in magnitude. Of the 18 subsets in which we test for the prevalence of *p-*hacking, we found consistent results (in both direction and significance) in nine of the datasets. Specifically, the critical bin comparisons indicated 12 positive effects (i.e., evidence for *p-*hacking), three of which were significant. Z-curve results indicated 11 positive effects, two of which were significant. For each case where we observe significance, we see that the effect size is in the direction of evidence for *p-*hacking (i.e., positive effect size). The differing findings of nine subsets across the two analytical approaches are perhaps due to the small magnitude of effect sizes. Considering Cohen’s [91] benchmarks of *g* (*h)* = .05 (.20), *g* = .15 (.50), and *g* = .25 (.80) as small, medium, and large, respectively, effect sizes observed in the present study should be considered negligible [77]. Thus, *p-*hacking may not be as large a problem to the cumulative knowledge of organizational research as some have cautioned in other areas of research [20–22,24–27,30–33].

Second, we set out to address the extent to which three predictors explained variance in *p-*hacking. Both analytical approaches were consistent in finding no relationship between authorship team size and *p-*hacking, thus, in contrast with previous research indicating that grouping may increase or decrease unethical behavior, we do not find support for either of these possibilities. Next, both approaches indicated that journal prestige had a positive relationship with *p-*hacking. In light of this consistent finding, researchers may wish to routinely test for potential moderation from journal prestige in future meta-analyses. Finally, the analytical approaches differed such that z-curve results indicated an increase in *p-*hacking in more recent decades, whereas the critical bin comparison approach indicated a decline in *p-*hacking over time. Importantly, none of the odds ratios for publication year differed from 1.0 significantly. These results remained mostly consistent with those of the 15 bivariate relation types. It is important to note that these approaches are not directly comparable because the regressions include control variables. However, after removing covariates and retaining only publication year as a predictor, we found similar results in the complete dataset (*OR* = .98, 95% CI [.96, 1.00], *p* = .115).

### Implications for future research

The present study offers implications for future research. Head et al. [13] found a larger effect for the prevalence of *p-*hacking in psychology and cognitive sciences (*g =* .133; close to Cohen’s medium benchmark, *g* = .15) than other scientific fields, although based on a very small sample of *p*-values. Our large-scale analysis provides a contrast–*g* = -.005, .006, .028 for the complete, substantive, and non-substantive datasets, respectively. Similarly, the z-curve yielded negligible to small effects (*h* = .151, .014, .163; all below Cohen’s .20 small benchmark) and indicates the prevalence of *p-*hacking in organizational psychology is much smaller and less concerning than previous researchers have suggested. One possible explanation for this difference is that *p-*hacking might be more common in experimental literatures than non-experimental literatures. Indeed, consider that experimental psychologists might be interested in a single, key effect (e.g., difference between two conditions; cf. [45]), whereas nonexperimental psychologists are likely interested in correlations among many variables. Thus, future research could examine differences in *p-*hacking between experimental and non-experimental organizational research or between zero-order and higher-order effects.

Importantly, however, meta-analysts in organizational research most often cull zero-order effects contained in correlation matrices. Thus, our cumulative evidence, insofar as it is revealed to us via meta-analysis and at least in the near-term, may not exposed to high risk from *p*-hacking. Indeed, meta-analyses are more often cited and more likely to reach practitioners and appear in undergraduate and graduate textbooks [35]. Although the two approaches yielded different results both in direction and significance for the complete metaBUS dataset, we remind the readers that these effect sizes, when interpreting according to Cohen’s benchmarks, range from negligible to small. We present these mixed results, however, with a greater deal of certainty that perhaps our cumulative knowledge is not exposed to great risk of *p-*hacking, which supports Lakens’ [83] assertion that the scientific literature is flawed due to reasons other than *p-*hacking (e.g., lack of statistical power, publication bias). Indeed, with the largest manually curated database available, our results indicate limited evidence of *p*-hacking. We assert this because we observed several small, significant positive effects (i.e., evidence of *p*-hacking) and no significant negative effects. Most often, however, the analyses revealed effects that failed to reach significance. This pattern is consistent with a true effect whose size is small or negligible.

As a rough approximation of hypothesis relevance likelihood, we classified the data into substantive versus non-substantive datasets. As a sort of manipulation check, comparisons of the two datasets indicate they behaved as intended. Indeed, as expected, the ODR (i.e., proportion of findings with *p* < .05) is greater in the substantive set. Similarly, as expected, substantive effects were larger in magnitude than non-substantive effects. Finally, the evidential value results (i.e., comparing lower- vs. upper-half of *p* < .05 region) indicate greater evidential value in the substantive set compared with the non-substantives set. However, what is puzzling about our results is that both approaches show a greater magnitude *p-*hacking in the non-substantive dataset than the substantive dataset. This is opposite of what we would expect such that these relations are less likely hypothesis-relevant and, thus, researchers should be *less* inclined to *p-*hack these effect sizes. This is an interesting avenue for future researchers to further disentangle and understand why these trends may occur.

Finally, as described earlier, our target sample size to reach .80 power for the critical bin comparisons, *N* = 705, was based on an analysis of Head et al.’s [13] effect size *g =* .047. We now know that this value is not appropriate for a corpus of mixed zero-order effects in organizational research. Moving forward, considering the non-substantive analysis revealed the largest effect *g* = .028, in the best-case scenario, future research will need 1,991 observations (i.e., number of *p*-values between .040 ≤ *p* < .050) to obtain .80 power in organizational research. Practically speaking, future research should carefully consider this large sample size needed in order to adequately study *p-*hacking using quantitative methods. Too often, meta-analyses in organizational psychology are done with too few observations and have insufficient power–this can even be the case in meta-science [78]. Still, it remains important to understand how to curb unethical research behavior. Thus, future research could explore the relevant psychological mechanisms that motivate researchers to *p-*hack using larger datasets or qualitative methods. For example, future research could build on Butler et al. [1] by employing a qualitative data collection (e.g., interviews, focus groups) to gain a deeper understanding of several potential research questions such as 1) Do psychological mechanisms such as rationalization or self-regulation play a role in engagement in QRPs? 2) Are personality traits or characteristics predictors of engagement in QRPs? Finally, 3) How do different data analysis procedures influence QRP engagement?

### Limitations and future directions

Our study makes an important contribution to organizational literature, but it is not without limitations. First, we did not reach .80 power in all of our critical bin comparisons and some sample sizes are smaller than the anticipated *N* = 705, thus, we focused on commonly studied bivariate relation types in organizational research to maximize sample size. However, as described above, the magnitude of *p*-hacking was frequently so minuscule that even the largest database of manually curated findings -with exact *p*-values- was, at times, underpowered. Moreover, at the alpha level of .05, we would expect one of every 20 findings to be significant, thus, in the present study, we would expect less than one of the 18 subsets to be significant. However, critical bin comparisons revealed three subsets with significant *p-*hacking and z-curve analyses revealed two. Future research could examine differences within specific bivariate relation types, assuming a sufficiently large sample could be amassed, or using different approaches to detect *p*-hacking. Nonetheless, for many of our analyses, we overcame limitations of previous research and provided a comprehensive analysis of the impact of *p*-hacking in cumulative knowledge using two analytical approaches in organizational research.

Second, our analysis does not account for cross-cultural differences. Indeed, it is possible that *p*-hacking might be more or less likely in some regions compared with others. Thus, future research could investigate *p*-hacking cross-culturally to explore variations by researchers’ country of origin or location of sample. Similarly, another predictor of *p-*hacking worthy of exploration is hypothesis relevance. Indeed, as described earlier, organizational researchers often report correlations between many variables (e.g., correlation matrix), but might focus on *p*-hacking only the key effects of interest. In the present study, we did not consider hypothesis relevance because the metaBUS database does not presently contain this information and because meta-analyses in organizational research rely on zero-order effects in the form of correlations in 90% of cases [35], many of which are hypothesis-irrelevant (e.g., 52% of effect sizes used in Judge et al.’s meta-analysis were non-hypothesized; [92]). However, we attempted to address this limitation by analyzing our data by bivariate relation type to maximize the likelihood of hypothesis relevance.

Finally, future researchers should continue to advance research on QRPs in organizational psychology to foster a more rigorous and credible science. It is also important for leaders in the field to consider their important role in curbing *p-*hacking behavior and advancing reliable findings. Journal editors could encourage open science practices and replication studies and reduce emphasis on theoretical novelty. Although we recognize this is unlikely to become a reality in short order, some journals have recently taken steps to reduce the focus on statistically significant findings. For example, *Strategic Management Journal* does not allow references to cutoff levels of statistical significance and has made a recent transition to the acceptance of replication studies [58]. In fact, a new journal has been introduced by the Southern Management Association, *Journal of Management Scientific Reports*, that accepts manuscripts centered around replication, generalizability, and theory testing and refinement. These steps will, hopefully, reduce some of the focus on obtaining significant *p-*values for the sake of publication.

## Conclusion

Considering recent concerns of questionable research practices in organizational psychology and the attention on the reproducibility crisis, we set out to explore the prevalence and predictors of *p-*hacking of zero-order correlations in organizational research. We advance that the prevalence of *p-*hacking is detectable but small in magnitude. We observed a consistent positive relationship between journal prestige and *p-*hacking, and no relationship between authorship team size and *p-*hacking. Thus, the effects of *p-*hacking in organizational research -at least as far as our cumulative, meta-analytic knowledge is concerned- may more a tempest in a teacup than a sky falling.

## Supporting information

S1 Fig *P-*hacking results using the z-curve (According to each bivariate relation type). (DOCX)

S1 Table Multilevel logit regression results for predictors of bin membership (According to each bivariate relation type). (DOCX)
