# Supplementary material for: Tempest in a teacup: An analysis of p-Hacking in organizational research
Source: PLoS One. 2023 Feb 24;18(2):e0281938. doi: 10.1371/journal.pone.0281938 (PMC9955613; doi:10.1371/journal.pone.0281938)
Supplement: S1 Fig — (DOCX) [file pone.0281938.s001.docx]

**S1 Figure. *P-*hacking results using the z-curve (According to each bivariate relation type).**

| **DEMO-ORG**  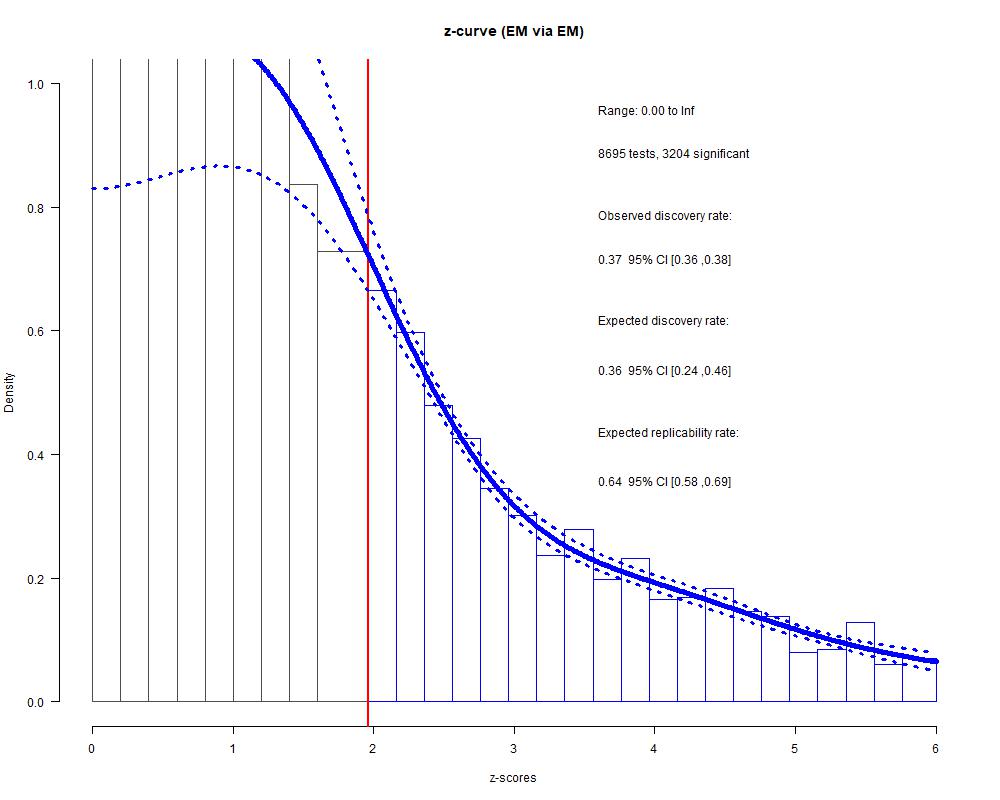 | **Attitudes-Attitudes**  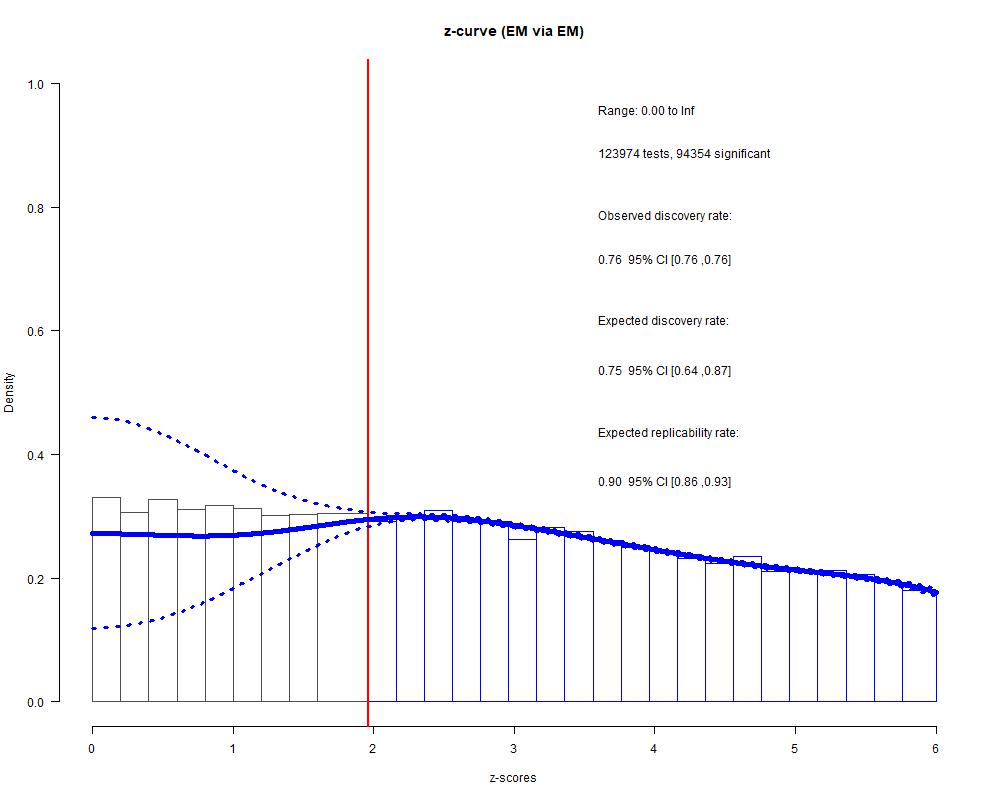 |
| --- | --- |
| **Attitudes-DEMO**  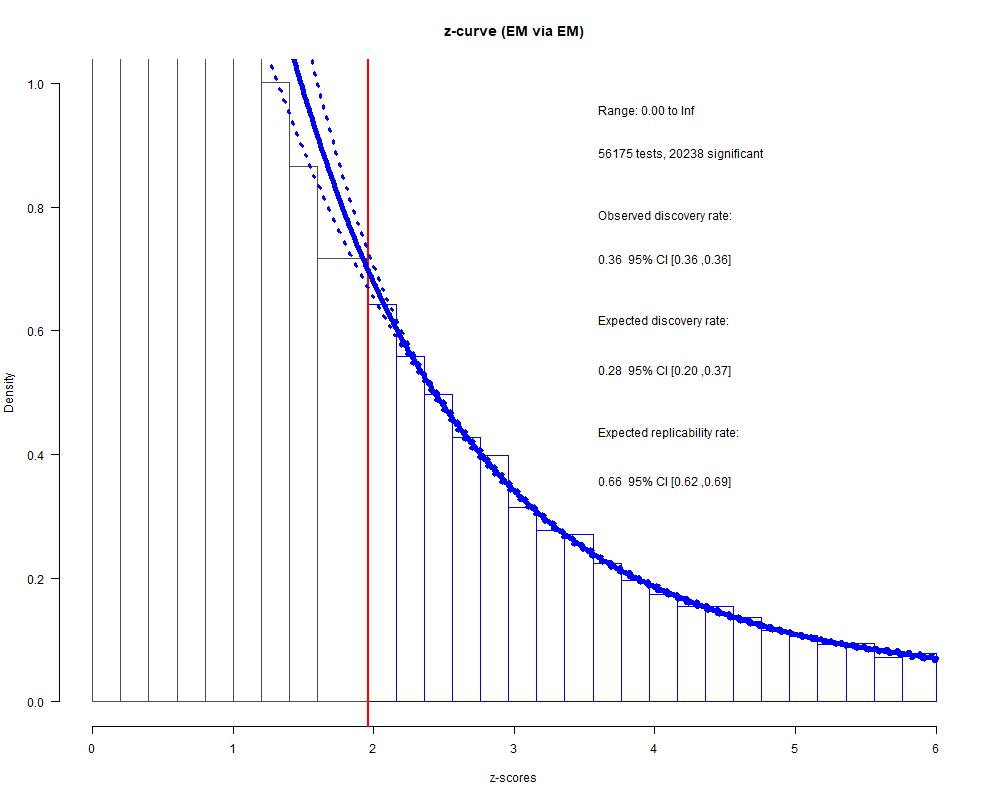 | **Attitudes-ORG**  **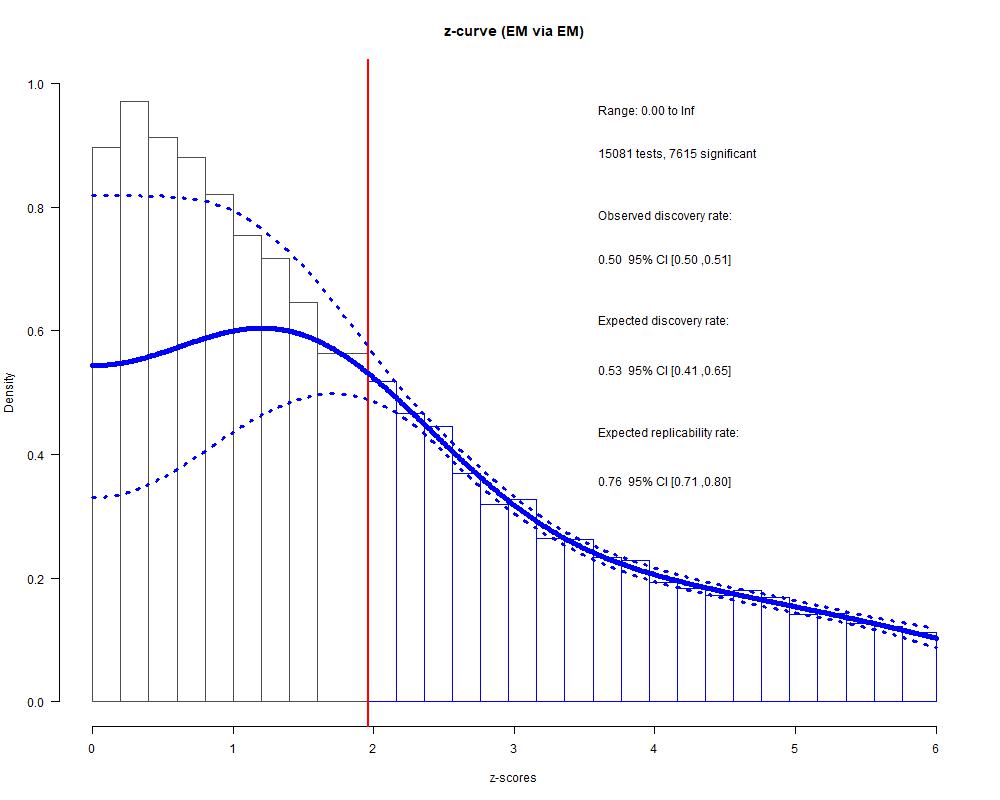** |
| **Behaviors-Behaviors**  **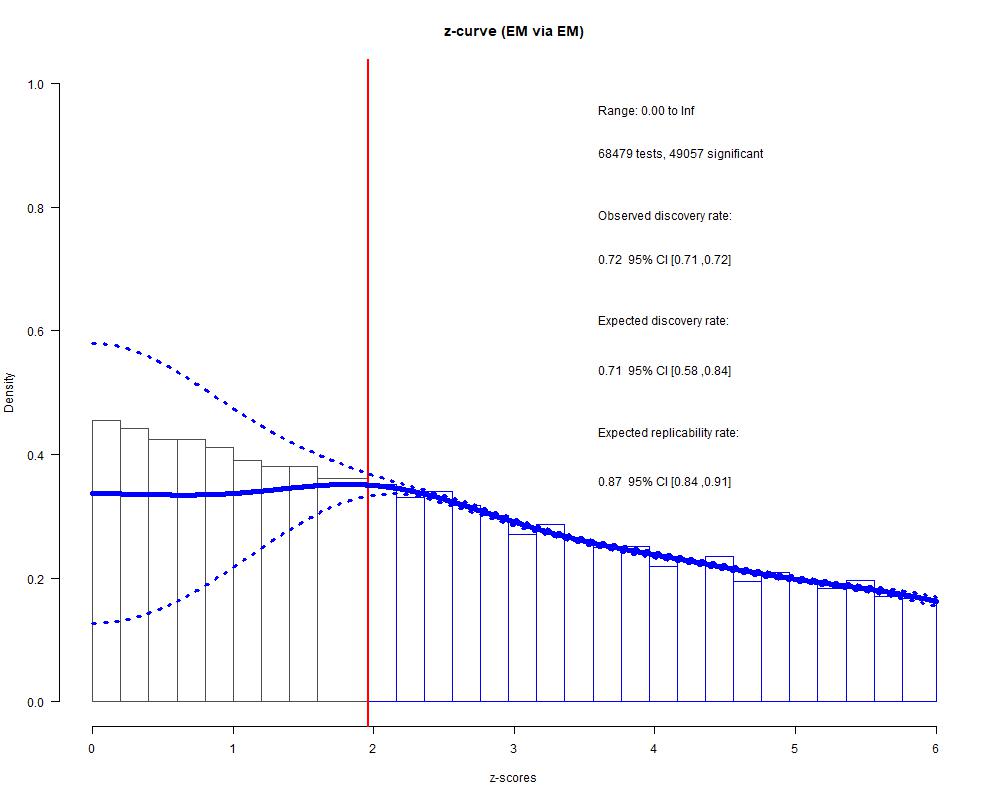** | **Behaviors-DEMO**  **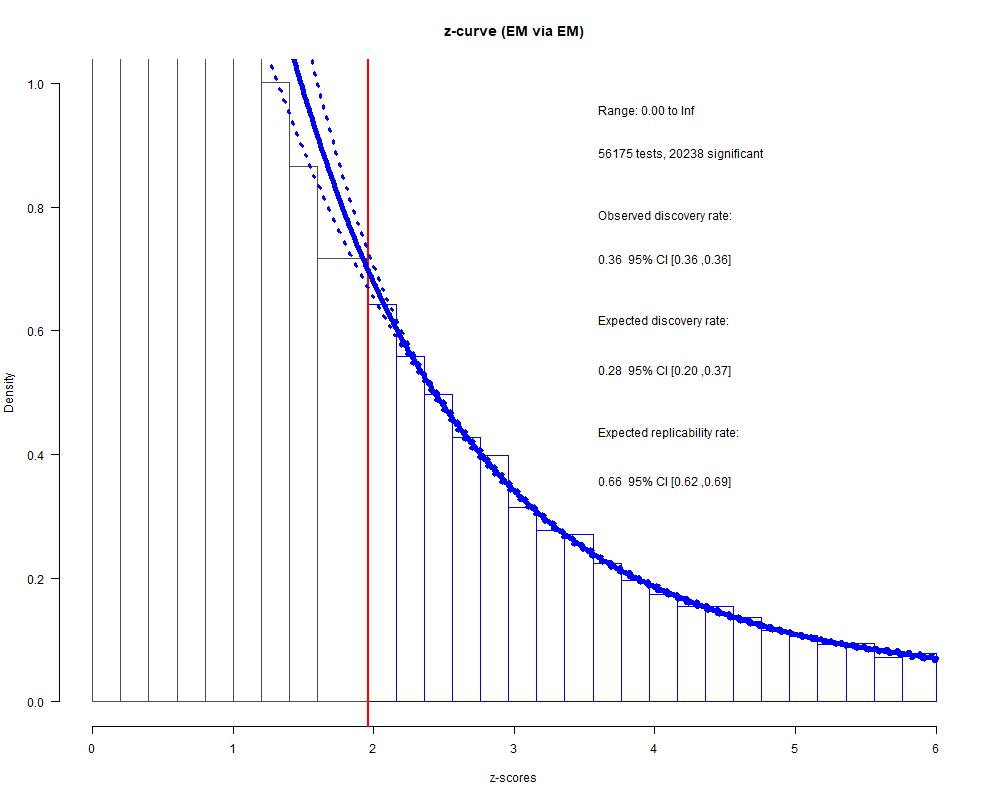** |
| **Behaviors-PSYC**  **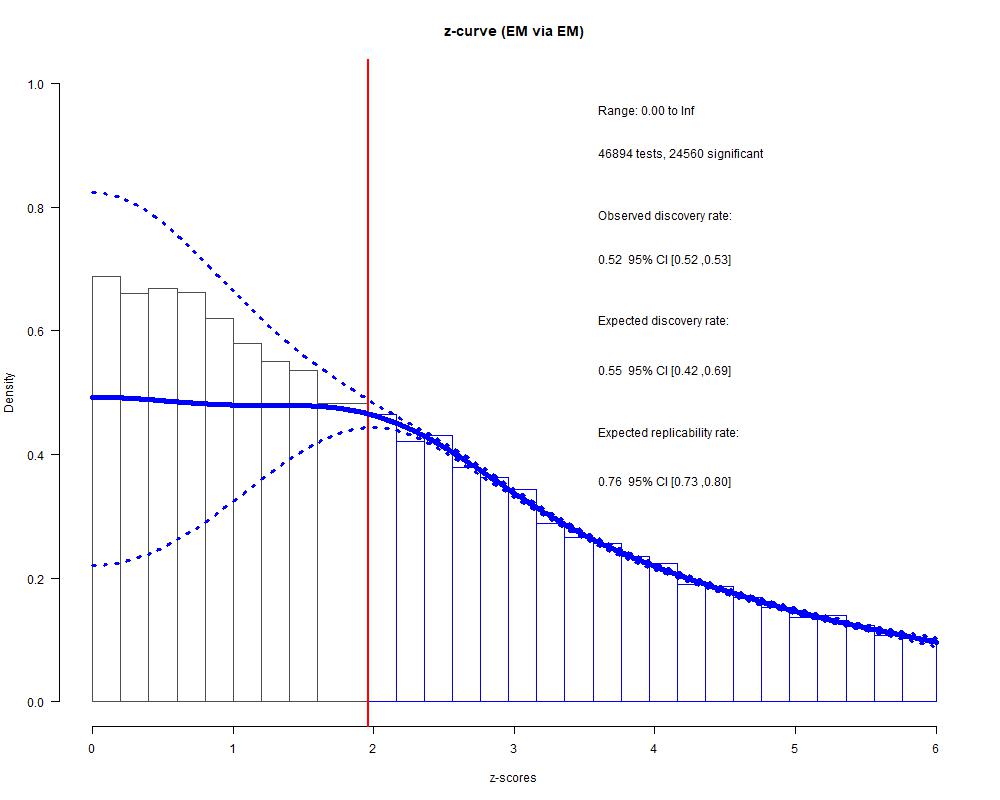** | **DEMO-DEMO**  **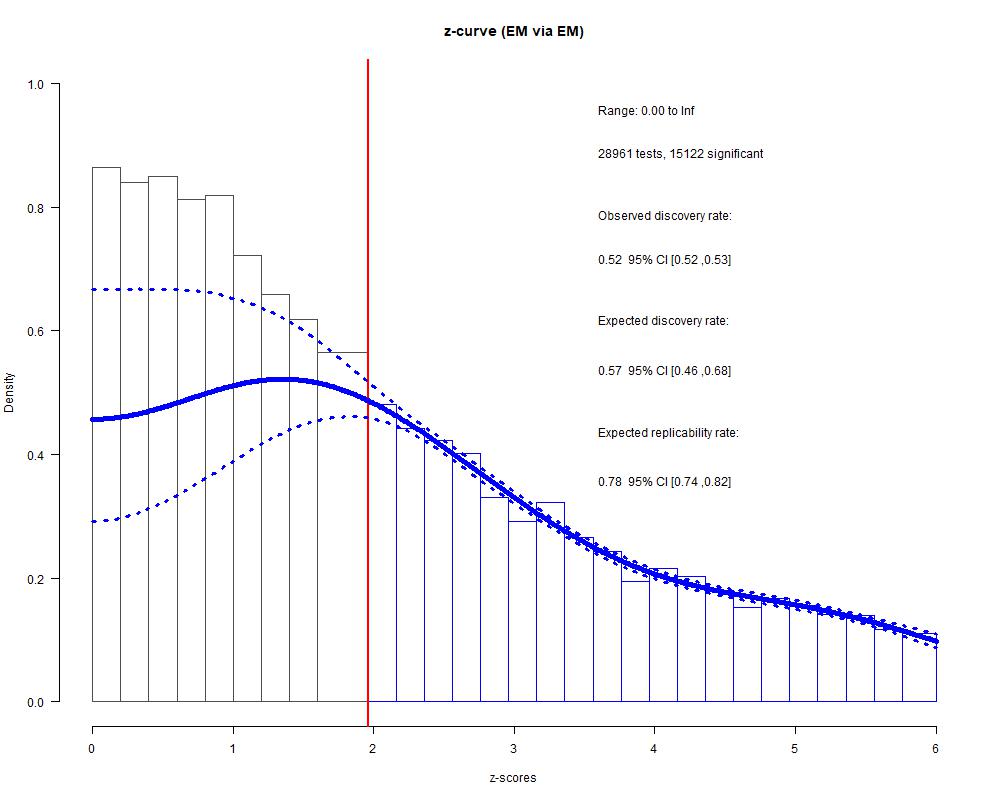** |
| **PSYC-DEMO**  **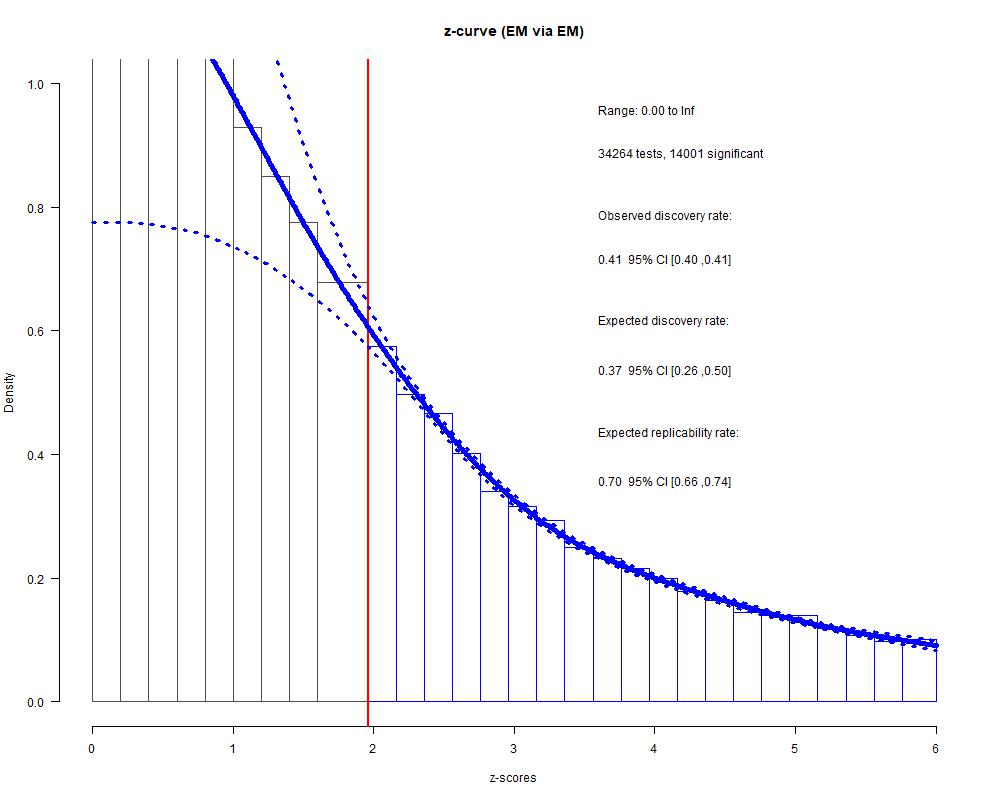** | **PSYC-ORG**  **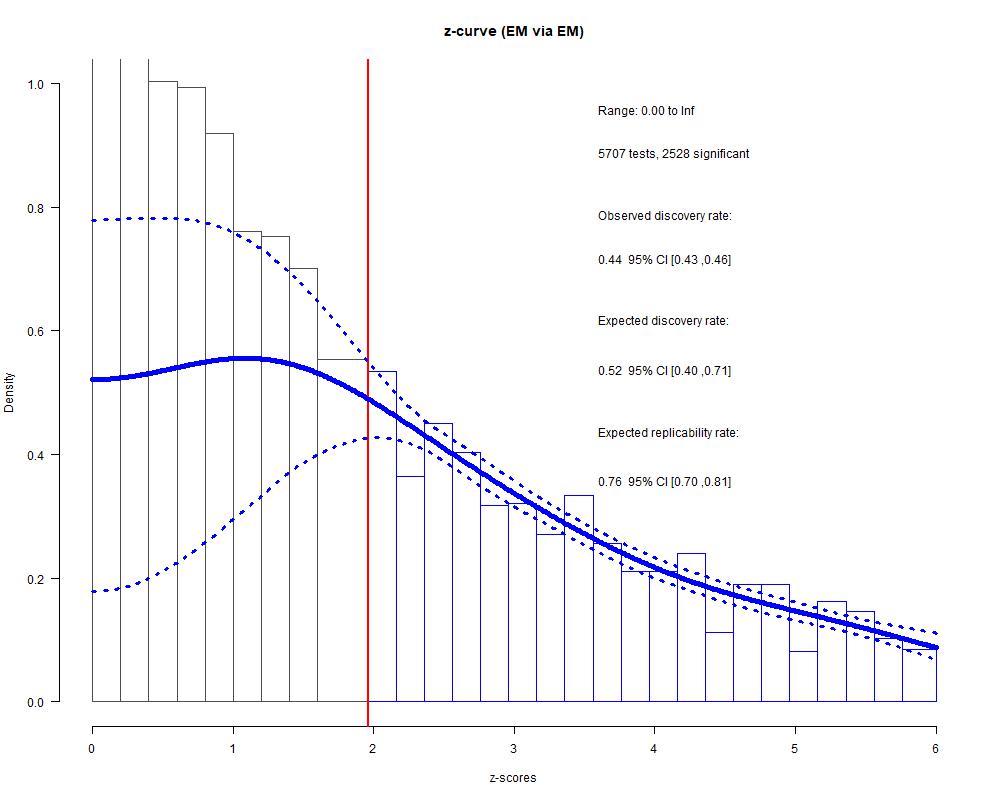** |
| **Attitudes-Behaviors**  **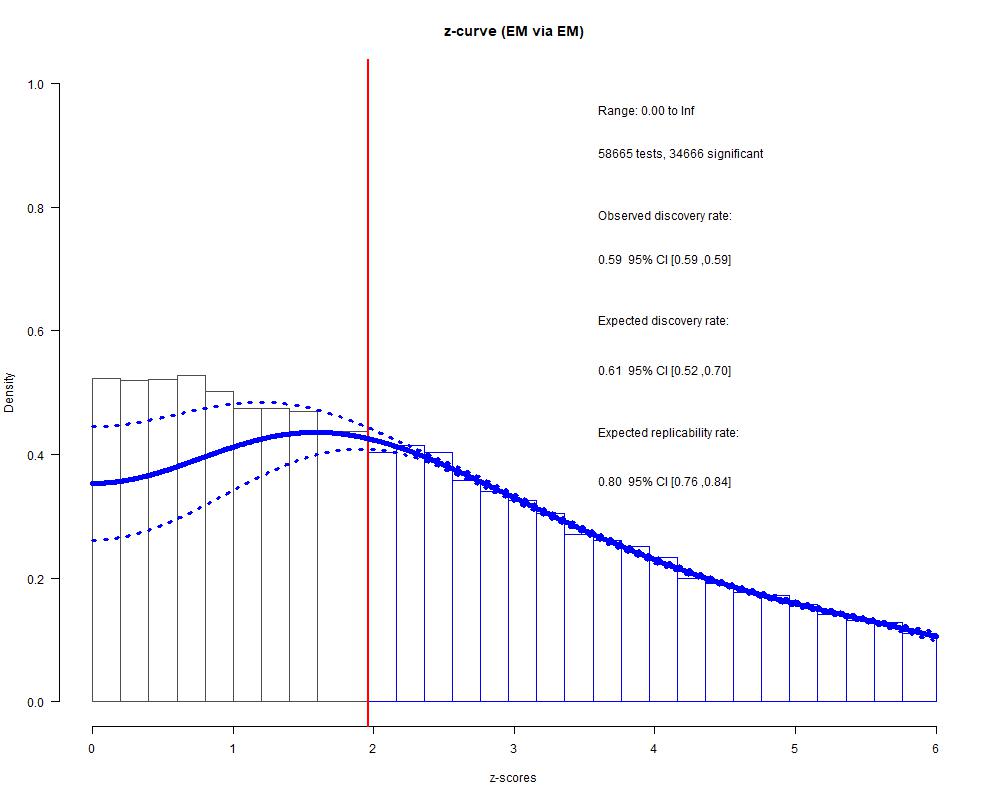** | **Attitudes-PYSC**  **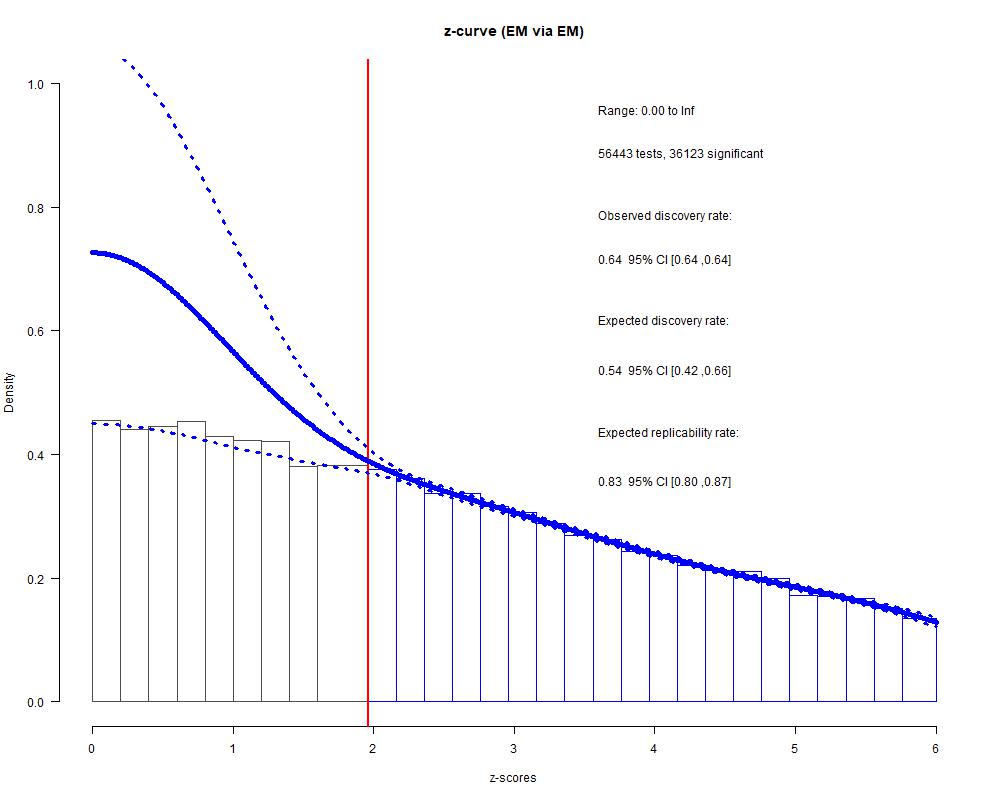** |
| **Behaviors-ORG**  **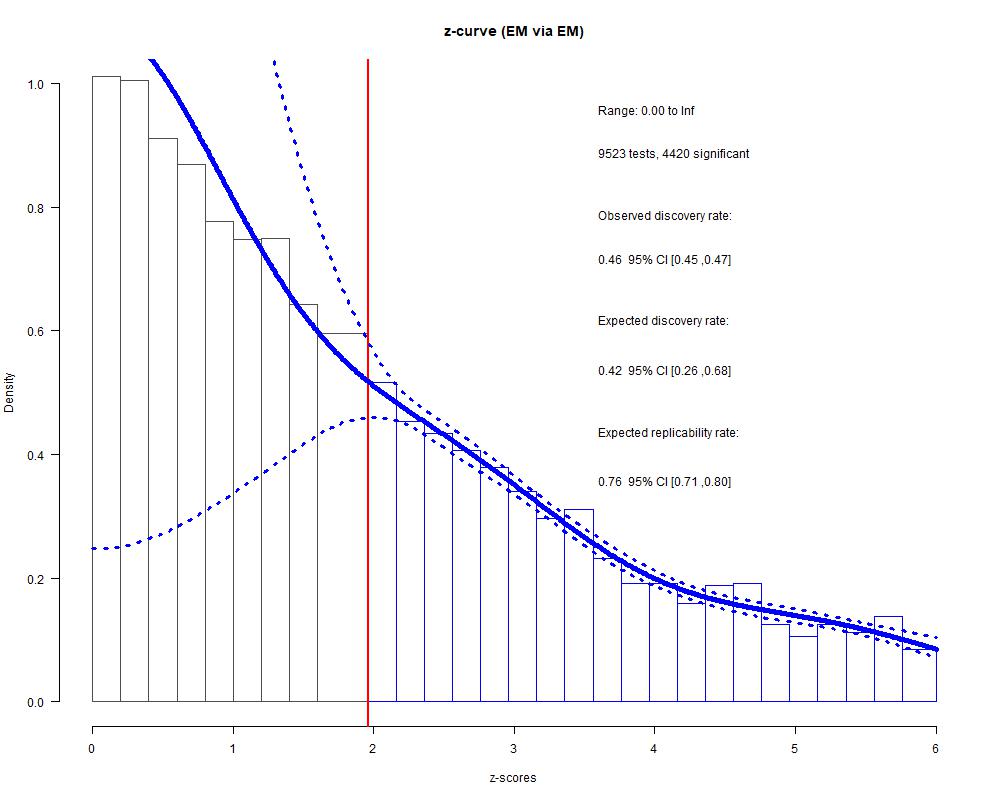** | **ORG-ORG**  **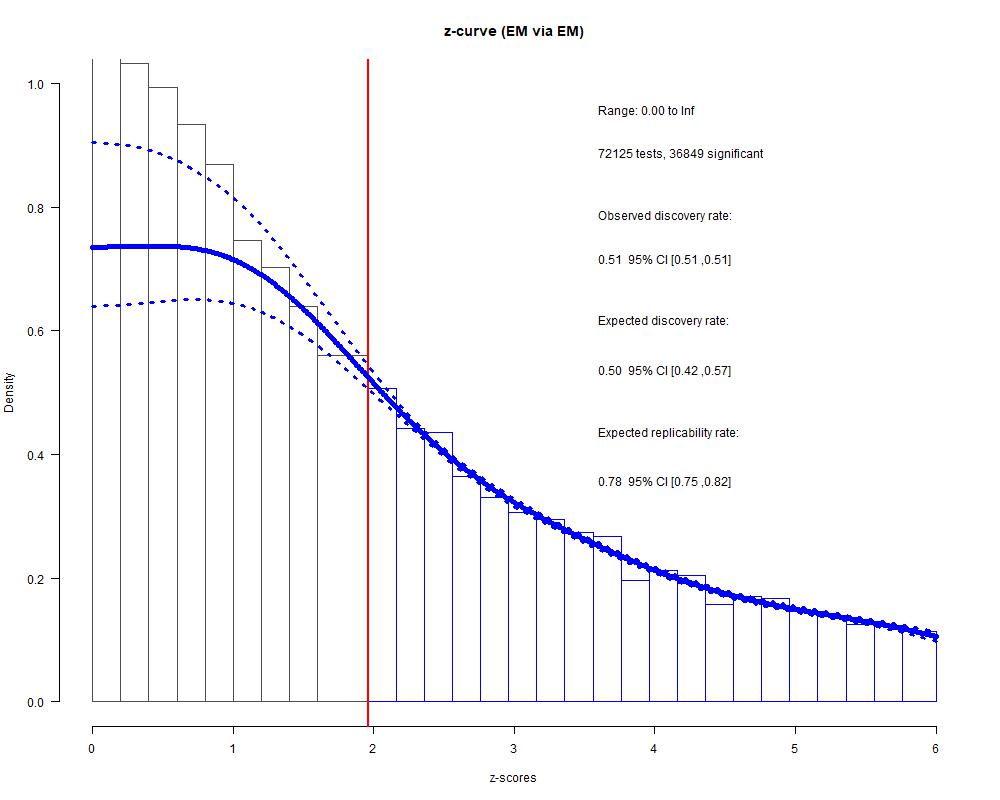** |
| **PSYC-PSYC**  **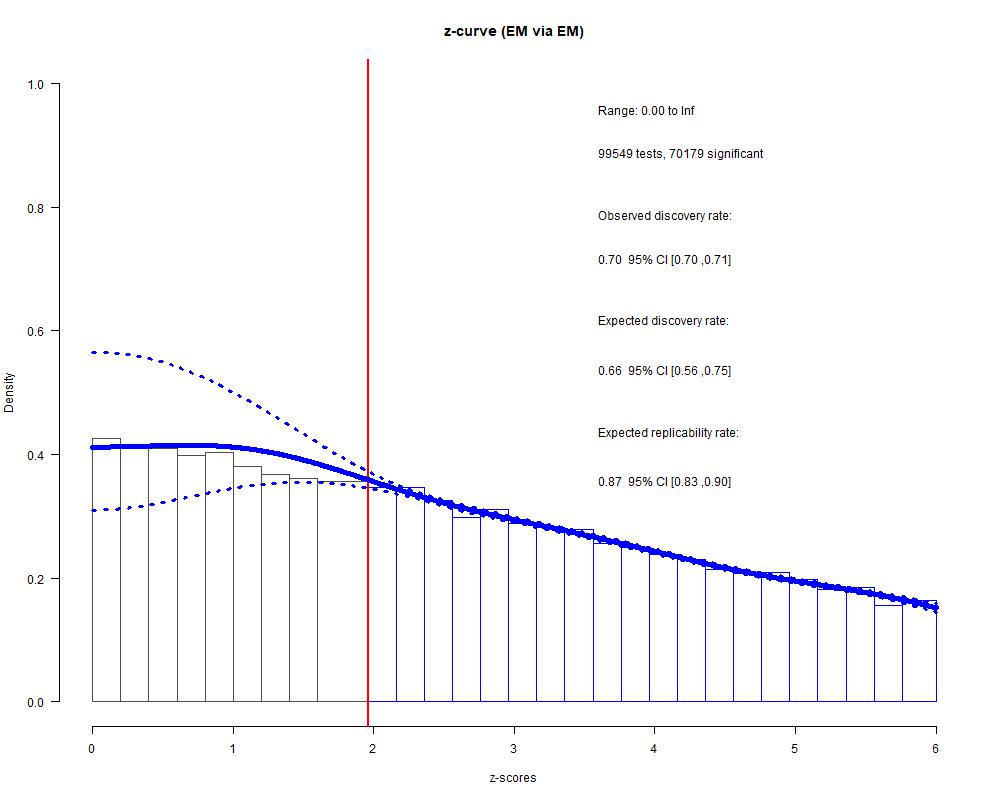** |  |

*Figure note.* PSYC = Psychological characteristics; DEMO = Objective person characteristics / demographics; ORG = Organizational characteristics. CI = Confidence interval. Z-curve analyses were conducted using expectation-maximization with 5,000 bootstrap samples.
