# Supplementary material for: Tempest in a teacup: An analysis of p-Hacking in organizational research
Source: PLoS One. 2023 Feb 24;18(2):e0281938. doi: 10.1371/journal.pone.0281938 (PMC9955613; doi:10.1371/journal.pone.0281938)
Supplement: S1 Table — (DOCX) [file pone.0281938.s002.docx]

**S1 Table. Multilevel logit regression results for predictors of bin membership (According to each bivariate relation type).**

| **Attitudes-Attitudes** | | | |  | **Attitudes-Behaviors** | | | |
| --- | --- | --- | --- | --- | --- | --- | --- | --- |
|  | **Bin Membership** | | |  |  | **Bin** | | |
| *Predictors* | *Odds Ratios* | *CI* | *p* |  | *Predictors* | *Odds Ratios* | *CI* | *p* |
| (Intercept) | 1.11 | 0.71 – 1.74 | 0.636 |  | (Intercept) | 1.1 | 0.75 – 1.62 | 0.635 |
| Journal Prestige | 1.39 | 0.88 – 2.20 | 0.162 |  | Journal Prestige | 1.49 | 0.98 – 2.29 | 0.065 |
| Publication Year | 0.59 | 0.36 – 0.97 | 0.037 |  | Publication Year | 0.9 | 0.60 – 1.35 | 0.62 |
| Authorship Team Size | 1.06 | 0.69 – 1.64 | 0.786 |  | Authorship Team Size | 1.26 | 0.84 – 1.88 | 0.266 |
| **Random Effects** | | | |  | **Random Effects** | | | |
| σ^2^ | 3.29 | | |  | σ^2^ | 3.29 | | |
| τ_00_ _Article_ | 16.45 | | |  | τ_00_ _Article_ | 10.7 | | |
| ICC | 0.83 | | |  | ICC | 0.76 | | |
| *N _Article_* | 722 | | |  | *N _Article_* | 621 | | |
| Observations | 1215 | | |  | Observations | 913 | | |
| Marginal R^2^ / Conditional R^2^ | 0.023 / 0.837 | | |  | Marginal R^2^ / Conditional R^2^ | 0.015 / 0.768 | | |
|  |  |  |  |  |  |  |  |  |
| **Attitudes-DEMO** | | | |  | **Attitudes-ORG** | | | |
|  | **Bin** | | |  |  | **Bin** | | |
| *Predictors* | *Odds Ratios* | *CI* | *p* |  | *Predictors* | *Odds Ratios* | *CI* | *p* |
| (Intercept) | 1.2 | 0.74 – 1.92 | 0.461 |  | (Intercept) | 0.92 | 0.41 – 2.07 | 0.845 |
| Journal Prestige | 1.09 | 0.68 – 1.76 | 0.708 |  | Journal Prestige | 2.25 | 0.77 – 6.55 | 0.138 |
| Publication Year | 0.81 | 0.50 – 1.30 | 0.375 |  | Publication Year | 0.90 | 0.38 – 2.13 | 0.818 |
| Authorship Team Size | 0.93 | 0.58 – 1.51 | 0.782 |  | Authorship Team Size | 1.39 | 0.62 – 3.14 | 0.424 |
| **Random Effects** | | | |  | **Random Effects** | | | |
| σ^2^ | 3.29 | | |  | σ^2^ | 3.29 | | |
| τ_00_ _Article_ | 15.28 | | |  | τ_00_ _Article_ | 11.55 | | |
| ICC | 0.82 | | |  | ICC | 0.78 | | |
| *N _Article_* | 635 | | |  | *N _Article_* | 155 | | |
| Observations | 962 | | |  | Observations | 208 | | |
| Marginal R^2^ / Conditional R^2^ | 0.004 / 0.824 | | |  | Marginal R^2^ / Conditional R^2^ | 0.050 / 0.789 | | |
| **Attitudes-PSYC** | | | |  | **Behaviors-Behaviors** | | | |
|  | **Bin** | | |  |  | **Bin** | | |
| *Predictors* | *Odds Ratios* | *CI* | *p* |  | *Predictors* | *Odds Ratios* | *CI* | *p* |
| (Intercept) | 0.92 | 0.41 – 2.07 | 0.845 |  | (Intercept) | 1.59 | 1.03 – 2.44 | 0.036 |
| Journal Prestige | 2.25 | 0.77 – 6.55 | 0.138 |  | Journal Prestige | 1.07 | 0.70 – 1.63 | 0.742 |
| Publication Year | 0.9 | 0.38 – 2.13 | 0.818 |  | Publication Year | 0.71 | 0.46 – 1.11 | 0.135 |
| Authorship Team Size | 1.39 | 0.62 – 3.14 | 0.424 |  | Authorship Team Size | 1.86 | 1.14 – 3.02 | 0.013 |
| **Random Effects** | | | |  | **Random Effects** | | | |
| σ^2^ | 3.29 | | |  | σ^2^ | 3.29 | | |
| τ_00_ _Article_ | 11.55 | | |  | τ_00_ _Article_ | 8.61 | | |
| ICC | 0.78 | | |  | ICC | 0.72 | | |
| *N _Article_* | 155 | | |  | *N _Article_* | 447 | | |
| Observations | 208 | | |  | Observations | 708 | | |
| Marginal R^2^ / Conditional R^2^ | 0.050 / 0.789 | | |  | Marginal R^2^ / Conditional R^2^ | 0.033 / 0.733 | | |
|  |  |  |  |  |  |  |  |  |
| **Behaviors-DEMO** | | | |  | **Behaviors-ORG** | | | |
|  | **Bin** | | |  |  | **Bin** | | |
| *Predictors* | *Odds Ratios* | *CI* | *p* |  | *Predictors* | *Odds Ratios* | *CI* | *p* |
| (Intercept) | 0.08 | 0.01 – 0.63 | 0.017 |  | (Intercept) | 0.68 | 0.25 – 1.87 | 0.46 |
| Journal Prestige | 63929.58 | 1517.44 – 2693345.67 | <0.001 |  | Journal Prestige | 1.42 | 0.54 – 3.74 | 0.479 |
| Publication Year | 0.25 | 0.03 – 1.83 | 0.173 |  | Publication Year | 1.37 | 0.51 – 3.72 | 0.532 |
| Authorship Team Size | 8.24 | 0.79 – 86.23 | 0.078 |  | Authorship Team Size | 0.78 | 0.30 – 2.01 | 0.60 |
| **Random Effects** | | | |  | **Random Effects** | | | |
| σ^2^ | 3.29 | | |  | σ^2^ | 3.29 | | |
| τ_00_ _Article_ | 815.48 | | |  | τ_00_ _Article_ | 10.16 | | |
| ICC | 1 | | |  | ICC | 0.76 | | |
| *N _Article_* | 350 | | |  | *N _Article_* | 105 | | |
| Observations | 452 | | |  | Observations | 145 | | |
| Marginal R^2^ / Conditional R^2^ | 0.141 / 0.997 | | |  | Marginal R^2^ / Conditional R^2^ | 0.015 / 0.759 | | |
| **Behaviors-PSYC** | | | |  | **DEMO-DEMO** | | | |
|  | **Bin** | | |  |  | **Bin** | | |
| *Predictors* | *Odds Ratios* | *CI* | *p* |  | *Predictors* | *Odds Ratios* | *CI* | *p* |
| (Intercept) | 0.63 | 0.34 – 1.15 | 0.132 |  | (Intercept) | 0.98 | 0.55 – 1.75 | 0.941 |
| Journal Prestige | 0.7 | 0.39 – 1.25 | 0.230 |  | Journal Prestige | 2.11 | 1.03 – 4.31 | 0.040 |
| Publication Year | 0.9 | 0.50 – 1.61 | 0.721 |  | Publication Year | 0.74 | 0.40 – 1.35 | 0.327 |
| Authorship Team Size | 1.02 | 0.53 – 1.96 | 0.944 |  | Authorship Team Size | 0.98 | 0.54 – 1.78 | 0.960 |
| **Random Effects** | | | |  | **Random Effects** | | | |
| σ^2^ | 3.29 | | |  | σ^2^ | 3.29 | | |
| τ_00_ _Article_ | 17.32 | | |  | τ_00_ _Article_ | 10.97 | | |
| ICC | 0.84 | | |  | ICC | 0.77 | | |
| *N _Article_* | 504 | | |  | *N _Article_* | 289 | | |
| Observations | 760 | | |  | Observations | 378 | | |
| Marginal R^2^ / Conditional R^2^ | 0.006 / 0.841 | | |  | Marginal R^2^ / Conditional R^2^ | 0.051 / 0.781 | | |
|  |  |  |  |  |  |  |  |  |
| **DEMO-ORG** | | | |  | **ORG-ORG** | | | |
|  | **Bin** | | |  |  | **Bin** | | |
| *Predictors* | *Odds Ratios* | *CI* | *p* |  | *Predictors* | *Odds Ratios* | *CI* | *p* |
| (Intercept) | 16619.33 | 967.03 – 285619.50 | <0.001 |  | (Intercept) | 0.43 | 0.09 – 1.97 | 0.275 |
| Journal Prestige | 1.37 | 0.24 – 7.87 | 0.724 |  | Journal Prestige | 1.16 | 0.40 – 3.38 | 0.782 |
| Publication Year | 1.26 | 0.13 – 12.29 | 0.844 |  | Publication Year | 0.32 | 0.05 – 2.18 | 0.245 |
| Authorship Team Size | 1.42 | 0.06 – 32.59 | 0.828 |  | Authorship Team Size | 0.56 | 0.21 – 1.48 | 0.242 |
| **Random Effects** | | | |  | **Random Effects** | | | |
| σ^2^ | 3.29 | | |  | σ^2^ | 3.29 | | |
| τ_00_ _Article_ | 1104.91 | | |  | τ_00_ _Article_ | 39.18 | | |
| ICC | 1 | | |  | ICC | 0.92 | | |
| *N _Article_* | 95 | | |  | *N _Article_* | 410 | | |
| Observations | 135 | | |  | Observations | 975 | | |
| Marginal R^2^ / Conditional R^2^ | 0.000 / 0.997 | | |  | Marginal R^2^ / Conditional R^2^ | 0.044 / 0.926 | | |
| **PSYC-DEMO** | | | |  | **PSYC-ORG** | | | |
|  | **Bin** | | |  |  | **Bin** | | |
| *Predictors* | *Odds Ratios* | *CI* | *p* |  | *Predictors* | *Odds Ratios* | *CI* | *p* |
| (Intercept) | 1.42 | 0.97 – 2.08 | 0.068 |  | (Intercept) | 0 | 0.00 – 0.00 | <0.001 |
| Journal Prestige | 1.15 | 0.80 – 1.64 | 0.451 |  | Journal Prestige | 0.89 | 0.05 – 16.71 | 0.940 |
| Publication Year | 1.03 | 0.72 – 1.49 | 0.857 |  | Publication Year | 0.83 | 0.07 – 10.48 | 0.883 |
| Authorship Team Size | 0.76 | 0.52 – 1.13 | 0.183 |  | Authorship Team Size | 1.01 | 0.06 – 17.00 | 0.996 |
| **Random Effects** | | | |  | **Random Effects** | | | |
| σ^2^ | 3.29 | | |  | σ^2^ | 3.29 | | |
| τ_00_ _Article_ | 4.95 | | |  | τ_00_ _Article_ | 887.45 | | |
| ICC | 0.6 | | |  | ICC | 1 | | |
| *N _Article_* | 390 | | |  | *N _Article_* | 46 | | |
| Observations | 516 | | |  | Observations | 60 | | |
| Marginal R^2^ / Conditional R^2^ | 0.010 / 0.605 | | |  | Marginal R^2^ / Conditional R^2^ | 0.000 / 0.996 | | |
|  |  |  |  |  |  |  |  |  |
| **PSYC-PSYC** | | | |  |  |  |  |  |
|  | **Bin** | | |  |  |  |  |  |
| *Predictors* | *Odds Ratios* | *CI* | *p* |  |  |  |  |  |
| (Intercept) | 0.71 | 0.39 – 1.30 | 0.273 |  |  |  |  |  |
| Journal Prestige | 1.25 | 0.70 – 2.26 | 0.452 |  |  |  |  |  |
| Publication Year | 0.89 | 0.49 – 1.61 | 0.693 |  |  |  |  |  |
| Authorship Team Size | 0.79 | 0.41 – 1.54 | 0.491 |  |  |  |  |  |
| **Random Effects** | | | |  |  |  |  |  |
| σ^2^ | 3.29 | | |  |  |  |  |  |
| τ_00_ _Article_ | 21.65 | | |  |  |  |  |  |
| ICC | 0.87 | | |  |  |  |  |  |
| *N _Article_* | 572 | | |  |  |  |  |  |
| Observations | 1225 | | |  |  |  |  |  |
| Marginal R^2^ / Conditional R^2^ | 0.006 / 0.869 | | |  |  |  |  |  |
| *Note.* Bin membership = 1 (.045 ≤ *p* < .050) or 0 (.040 ≤ *p* < .045); CI = 95% Confidence Interval; ICC = Intraclass correlation coefficient; σ2 = Residual variance; τ00 = Variance due to nesting (i.e., article characteristics). PSYC = Psychological characteristics; DEMO = Objective person characteristics / demographics; ORG = Organizational characteristics. Marginal R^2^ is calculated according to Johnson (2014) and indexes the variance associated with fixed factors; conditional is variance attributed to fixed and random effects combined. | | | | | | | | |
